# Lung Cancer Survivorship: Challenges and Care Needs

**DOI:** 10.3390/cancers18172856

**Published:** 2026-09-03

**Authors:** Massimiliano Cani, Paolo Cotogni, Alessandra Greco, Giacomo Ronconi, Elisa Lombardi, Matteo Fracchiolla, Stefania Vallone, Maria Vittoria Pacchiana, Irene Capizzi, Valentina Bertaglia, Simona Carnio, Luisella Righi, Maurizio Balbi, Lorenzo Belluomini, Paolo Bironzo, Silvia Novello

**Affiliations:** 1 Department of Oncology, University of Turin, 10043 Orbassano, Italy; 2 Gustave Roussy Cancer Center, Paris-Saclay University, 94800 Villejuif, France; 3 Pain Management and Palliative Care, Department of Anesthesia, Intensive Care and Emergency, Molinette Hospital, University of Turin, 10126 Turin, Italy; 4 Women Against Lung Cancer in Europe, 10043 Orbassano, Italy; 5 Dietetics and Clinical Nutrition Unit, San Luigi Gonzaga University Hospital, 10043 Orbassano, Italy; 6 Pathology Unit, Department of Oncology, University of Turin, 10043 Orbassano, Italy; 7 Radiology Unit, Department of Oncology, University of Turin, 10043 Orbassano, Italy; 8 Section of Oncology, Department of Engineering for Innovation Medicine (DIMI), University of Verona School of Medicine and Verona University Hospital Trust, 37134 Verona, Italy

**Keywords:** survivorship, toxicities, palliative care, care needs, lung cancer

## Abstract

Advances in lung cancer treatment have progressively improved survival across all disease stages. Some patients now achieve long-term survival, while others may remain on treatment for several years, particularly with immune checkpoint inhibitors and selected tyrosine kinase inhibitors. These advances have increased recognition of the survivorship needs of patients with lung cancer, including persistent symptoms, treatment-related toxicities, and social, psychological, and caregiver-related concerns. The integration of palliative care has also become increasingly relevant, reflecting a shift in the traditional care paradigm by extending supportive approaches beyond the end-of-life setting to patients living long-term with active disease. However, evidence and care models addressing lung cancer survivorship remain fragmented and focus predominantly on patients treated with curative intent. This narrative review provides a comprehensive overview of survivorship in lung cancer, from screening and early-stage to locally advanced and metastatic disease, while also examining more cross-cutting physical, psychological and social domains. Greater recognition and systematic assessment of these needs is essential to develop structured, coordinated, and personalized survivorship pathways for the growing and clinically heterogeneous population of individuals living with and beyond lung cancer.

## Simple Summary

Thanks to advances in lung cancer diagnosis and treatments, many people now live longer after a diagnosis, and some remain in therapy for several years. As survival improves, care must extend beyond controlling the tumor to address persistent symptoms, treatment-related side effects, and physical, emotional, social, and financial needs. These challenges may arise at any stage of the disease and can also affect family members and caregivers. This narrative review summarizes current knowledge on survivorship in people living with and beyond lung cancer, from screening and curative treatment to advanced disease and long-term therapy. It also examines key aspects of daily life, including physical activity, nutrition, psychological support, smoking cessation, financial difficulties, and caregiver needs. The aim is to help clinicians identify these concerns earlier and develop more structured, coordinated, and personalized models of long-term care.

## 1. Introduction

Until the early 2010s, treatment options for lung cancer were largely restricted to chemotherapy, with targeted options only beginning to emerge. Advances in diagnostics, imaging, and molecular profiling have since enabled earlier detection and a more precise characterization of tumor biology, supporting the development of increasingly personalized treatment strategies, including molecularly targeted therapies, particularly tyrosine kinase inhibitors (TKIs), as well as immune checkpoint inhibitors (ICIs), with clinical research in lung cancer now moving toward more complex therapeutic approaches, including antibody–drug conjugates (ADCs), bispecific antibodies, and vaccine-based strategies [1,2].

These advances have contributed to meaningful changes in the prognosis and clinical course of lung cancer: the 5-year relative survival rate for patients with lung cancer has progressively increased, reaching approximately 28% in the United States for patients diagnosed between 2015 and 2021 [3]. Moreover, in selected patients, modern treatments may provide durable disease control and prolonged survival even in the metastatic setting, as observed with ICIs and some TKIs [4,5].

As patients live longer after a lung cancer diagnosis, their needs increasingly extend beyond tumor control alone. Long-term treatment-related toxicities, persistent symptoms, psychological distress, social and financial consequences, and the effects of cancer on caregivers have therefore become increasingly relevant components of care.

The National Coalition for Cancer Survivorship (NCCS) defines cancer survivorship as the period extending from diagnosis through the remainder of an individual’s life. Although the term primarily refers to the person diagnosed with cancer, the survivorship experience also encompasses caregivers and others whose lives are affected by the disease and its consequences [6].

The changing clinical trajectory of lung cancer has thus broadened the relevance of survivorship. At the same time, palliative care remains an essential and complementary component of management across disease stages, addressing symptoms, quality of life, and supportive needs. Despite this growing interest, survivorship research and care models have traditionally focused on patients with early-stage disease treated with curative intent, whereas the needs of patients with locally advanced or metastatic disease remain less extensively investigated and inconsistently addressed [7,8].

This review will examine the key features of survivorship and palliative care in lung cancer across different disease settings, including early-stage, locally advanced, and metastatic disease, as well as broader aspects of survivorship care and their impact on daily life across physical, psychological, social, and financial domains.

## 2. Methods

This narrative review provides a clinically oriented overview of lung cancer survivorship, based on a literature search conducted across PubMed/MEDLINE, Scopus, and Web of Science, using combinations of terms related to lung cancer, survivorship, long-term survival, screening, treatment-related toxicities, supportive and palliative care. No predefined date restrictions were applied, and the literature search was last updated in July 2026.

Relevant original studies, including clinical trials, observational studies, as well as systematic reviews and meta-analyses, clinical guidelines, and consensus or position documents, were considered according to the clinical topics addressed. The topics and evidence included in the manuscript were selected by consensus among the authors based on their relevance. Evidence was qualitatively categorized based on the literature reviewed, without applying a formal evidence-grading system. Language and terminology were aligned with the recommendations of the International Association for the Study of Lung Cancer Language Guide [9].

## 3. Lung Cancer Survivorship Across Disease Stages

**Figure 1:**
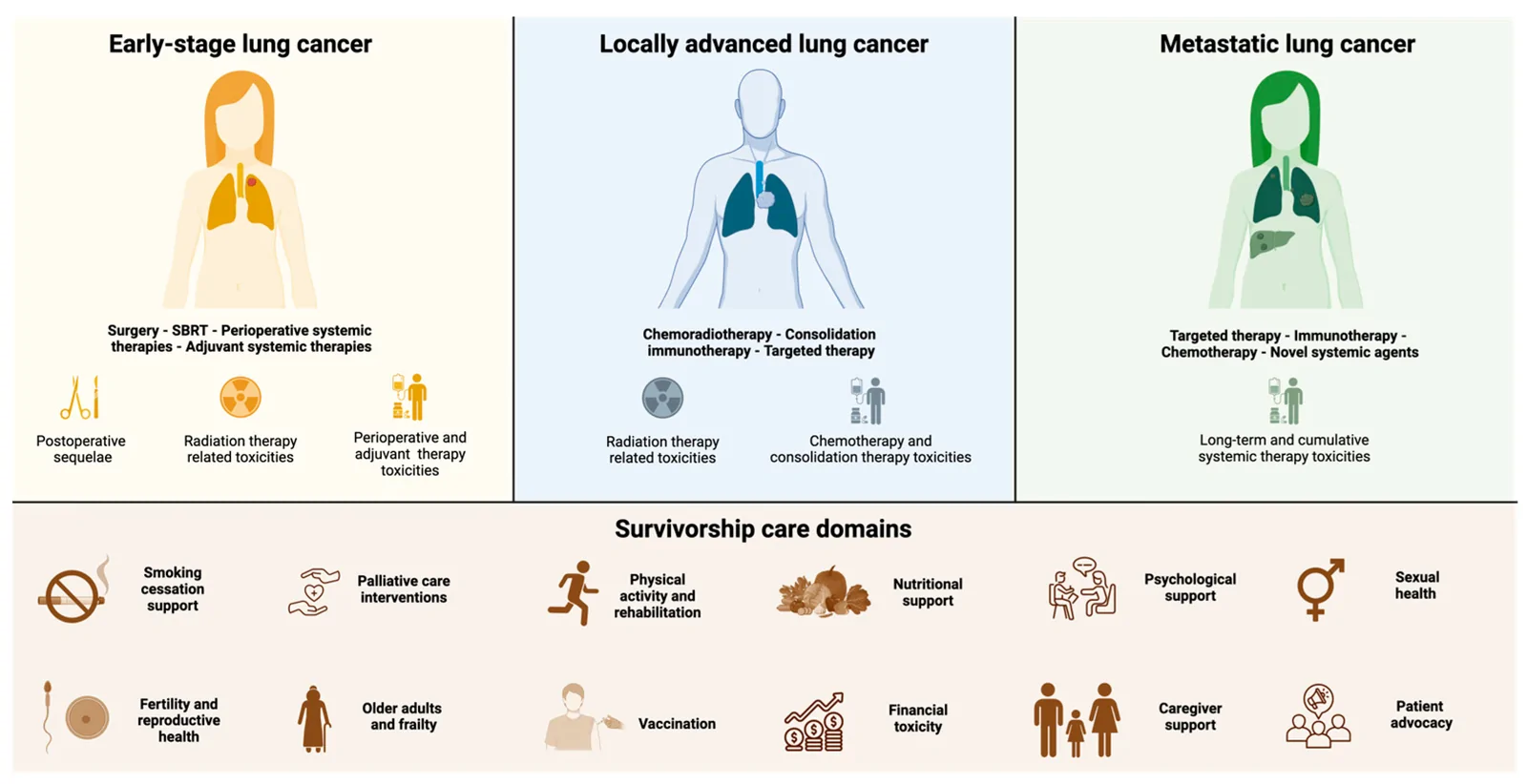
Stage-specific treatment exposures and cross-cutting domains of lung cancer survivorship care.

The following sections explore survivorship across different clinical settings and disease stages, with particular attention to treatment-related effects that may influence long-term care and their implications for surveillance and management (Figure 1).

### 3.1. Screening and Survivorship Implications

Although lung cancer screening with low-dose computed tomography (LDCT) precedes survivorship, it has important implications for subsequent survivorship care, as it increases the proportion of cancers detected at earlier stages, thereby expanding the population of potential long-term survivors, while also providing an early opportunity to address tobacco dependence and smoking-related comorbidities (e.g., lung emphysema and coronary calcifications) [10,11,12,13].

Indeed, lung cancer screening programs have been shown to reduce lung cancer-specific mortality, and, to a lesser extent, overall mortality among high-risk individuals with a history of tobacco consumption [14,15], providing evidence to implement lung cancer screening in international guidelines [16,17,18].

Following completion of the initial guideline-recommended surveillance period after curative-intent treatment, generally based on diagnostic CT imaging, LDCT may also provide a suitable strategy for longer-term surveillance in high-risk patients. In this setting, its main role is the detection of second primary lung cancers, whose risk persists beyond the period in which disease recurrence is most likely to occur [19]. However, evidence supporting the optimal use of LDCT in lung cancer survivors remains limited, partly because eligibility criteria, imaging protocols, and follow-up schedules have been heterogeneous across screening trials and survivorship studies [20].

In this context, minimizing cumulative radiation exposure becomes particularly relevant [21]. Imaging protocols with lower radiation exposure, including ultra-low-dose CT, are being evaluated or implemented in selected settings, supported by advances in artificial intelligence-based reconstruction algorithms [22,23]. Similarly, the interval between CT assessments may be tailored according to individual risk [24,25].

Furthermore, complementary methods, such as liquid biopsy using analytes like microRNA, circulating tumor cells, or circulating tumor DNA, and metabolic risk signatures, can better define a risk profile for personalized assessment or even help organize follow-up more effectively. The evidence in this context, however, remains limited, and the clinical applicability of these methods remains to be fully determined [25,26]. Beyond lung cancer screening, participation in other established cancer screening programs, including breast, cervical, and colorectal cancer screening, should be encouraged in accordance with local recommendations and tailored to age, sex, individual risk, overall health status, and life expectancy, particularly among patients treated with curative intent and those with advanced disease who achieve prolonged survival [27]. This broader preventive approach is particularly relevant given the persistent risk of subsequent primary malignancies among lung cancer survivors.

### 3.2. After Curative-Intent Treatment

Surgery remains the preferred curative-intent treatment for early-stage disease whenever feasible, while radiation therapy (e.g., Stereotactic Body Radiation Therapy, SBRT) represents a valid alternative [16,18].

The therapeutic landscape of resectable non-small-cell lung cancer (NSCLC) has substantially evolved in recent years. Beyond cisplatin-based adjuvant chemotherapy, adjuvant immunotherapy (IMpower010 trial) and targeted therapies for molecularly defined subgroups are now recommended [28,29,30], while ICIs have moved into the neoadjuvant and perioperative settings [31,32,33]. As a result, a growing proportion of patients treated with curative intent are now exposed to prolonged or multimodal systemic treatment, making the evaluation of late toxicities and long-term outcomes an essential part of their care.

Among survivors, pain, fatigue, dyspnea, emotional distress, and fear of recurrence are among the most frequent and persistent concerns [34]. These outcomes may be influenced by both modifiable factors and treatment-related burden. Among patients who were smoking at the time of lung cancer diagnosis, approximately half continue to smoke thereafter. Continued smoking and pre-existing comorbidities have been associated with poorer survival and health-related quality of life, whereas more extensive surgical resection and adjuvant systemic treatment may adversely affect postoperative recovery [35,36,37]. Pneumonectomy, in particular, is associated with worse physical functioning and reduced respiratory reserve compared with less extensive resections, supporting parenchyma-sparing approaches whenever oncologically adequate [38]. In addition, chronic post-thoracotomy pain remains a well-recognized late effect that may persist even after minimally invasive procedures [39].

**Figure 2:**
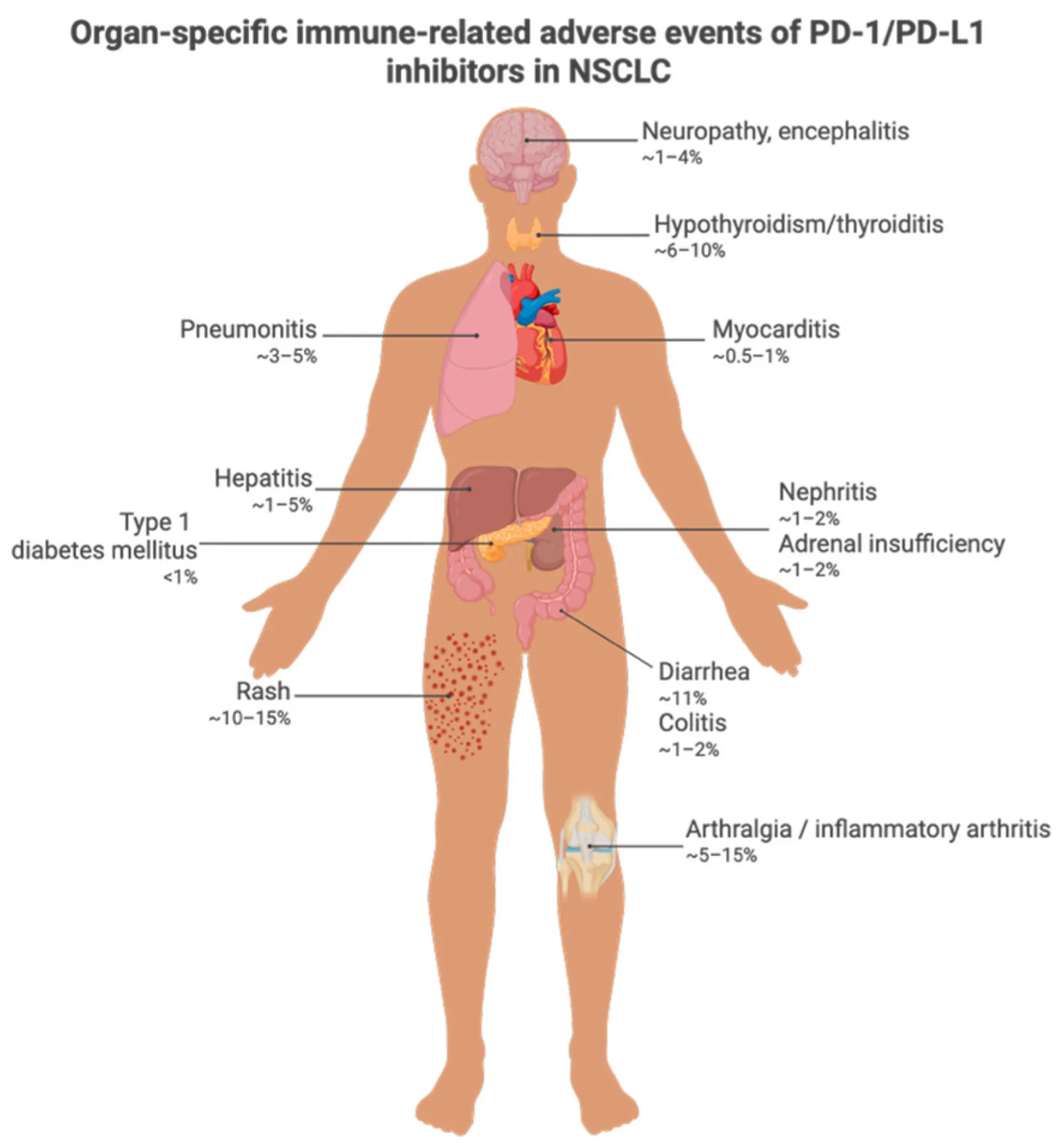
Organ-specific immune-related adverse events of PD-1/PD-L1 inhibitors in NSCLC.

Systemic treatment adds further long-term toxicities. Cisplatin-based chemotherapy may cause persistent sensory neuropathy and hearing loss [40], while the long-term survivorship impact of perioperative immunotherapy remains insufficiently characterized, particularly considering persistent immune-related adverse events and endocrine sequelae [41,42] (Figure 2).

Post-treatment follow-up after curative-intent therapy for early-stage NSCLC should therefore extend beyond recurrence surveillance to address persistent symptoms, late toxicities, functional and psychological recovery, smoking cessation, and comorbidities. It should be personalized according to clinical, pathological, molecular, functional, and psychosocial factors, as well as individual needs. Survivorship issues related to radiotherapy are discussed in the following section.

### 3.3. Long-Term Outcomes After Chemoradiotherapy

For patients with unresectable stage III NSCLC, definitive chemoradiotherapy represents the standard curative-intent strategy [16]. In this setting, the PACIFIC trial established consolidation durvalumab after platinum-based chemoradiotherapy as a standard of care, demonstrating a sustained overall survival (OS) benefit [43,44].

In this same context, the phase III LAURA trial demonstrated a significant progression-free survival (PFS) advantage with osimertinib after definitive chemoradiotherapy in patients with unresectable stage III *EGFR*-mutated NSCLC without disease progression, supporting its integration into current treatment algorithms [45].

As in early-stage disease, improvements in disease control and survival in this setting have increased attention to survivorship outcomes. However, patients treated with definitive chemoradiotherapy may face distinct challenges. In this context, common short-term toxicities include esophagitis, hematologic toxicity, and additional regimen-specific adverse events such as neuropathy and alopecia, mainly with taxane or etoposide-based schemes [46,47].

Beyond these acute effects, other toxicities may persist over time or emerge later and substantially affect long-term outcomes. Among these, pulmonary toxicity represents one of the most clinically relevant survivorship concerns after thoracic chemoradiotherapy, especially in patients with pre-existing smoking-related lung disease [48].

In the PACIFIC phase III trial, grade 3–4 pneumonitis occurred in 4.4% of patients treated with durvalumab, with higher rates reported in some real-world analyses, suggesting that pulmonary toxicity may result from the combined effects of thoracic radiotherapy and consolidation immunotherapy [43,49,50,51]. A similar overlap in pulmonary toxicity was also observed in the LAURA trial in which radiation pneumonitis occurred in 48% of patients receiving osimertinib compared with 38% receiving placebo [45].

Diagnosis of radiation-induced lung injury requires exclusion of alternative causes, while management depends on severity and may include corticosteroids, supportive respiratory care, rehabilitation, bronchodilators, and oxygen therapy. Although modern radiotherapy techniques can reduce exposure of healthy lung tissue, pulmonary toxicity remains dose-dependent, and treatment options for advanced fibrosis are limited, with antifibrotic agents still under investigation [52,53].

Cardiovascular toxicity is another major component of long-term survivorship after thoracic chemoradiotherapy [54]. Thoracic irradiation and platinum-based chemotherapy may contribute to endothelial dysfunction, accelerated atherosclerosis, myocardial fibrosis, vascular injury, arrhythmias, heart failure, valvular disease, and pericardial complications [55]. These risks are relevant in lung cancer survivors, who often have shared cardiovascular risk factors, including tobacco exposure, older age, hypertension, diabetes, and pre-existing cardiopulmonary disease [56]. Baseline cardiovascular assessment, optimization of modifiable risk factors, and referral to cardio-oncology services when clinically indicated should be considered part of survivorship care [57].

Furthermore, ICIs may cause immune-mediated cardiovascular toxicities, including myocarditis and pericardial disease. Although uncommon, these events can be severe and may present with nonspecific symptoms such as dyspnea, chest pain, fatigue, or palpitations; a prompt evaluation is thus essential [57].

Overall, survivorship care after definitive chemoradiotherapy should integrate respiratory and cardiovascular monitoring, management of persistent symptoms, rehabilitation strategies, prevention of infectious complications, and early recognition of immune-related toxicities.

### 3.4. Living with Advanced Disease

More than 70% of lung cancers are diagnosed with regional or distant disease, including approximately half with distant metastases. In NSCLC, particularly adenocarcinoma, treatment selection is largely determined by the tumor molecular profile and by biomarkers such as programmed death-ligand 1 (PD-L1) expression [58]. Improvements in systemic therapy have led to a growing proportion of patients achieving durable disease control, making survivorship relevant even in the metastatic setting. Unlike survivorship after curative-intent treatment, which is primarily focused on recurrence surveillance, late treatment effects, and recovery, survivorship in advanced disease occurs alongside active anticancer treatment and therefore requires continued monitoring and management of treatment-related toxicities and symptoms, with particular attention to functional preservation and quality of life. Although no consistent definition has been established, long-term responders have generally been described as patients maintaining an objective response or stable disease for at least 24 months (PFS ≥ 24 months) [59] or surviving for more than 3 years [60]. In this population, treatment-related toxicities may persist or accumulate over time, depending on the agent administered and the duration of exposure.

The acute and potentially persistent toxicities of the main targeted agents are summarized in Table 1.

In patients with NSCLC harboring common *EGFR* mutations, the FLAURA2 and MARIPOSA trials showed improved efficacy outcomes with osimertinib plus platinum–pemetrexed and amivantamab plus lazertinib, respectively, compared with osimertinib monotherapy, although these benefits were accompanied by greater toxicity and treatment burden [61,62]. For amivantamab-based therapy, proactive dermatologic prophylaxis (including clindamycin 1% on scalp, chlorhexidine 4% on fingernails and toenails, and ceramide-based moisturizer on body and face as proven in the COCOON trial) can reduce clinically relevant cutaneous adverse events, whereas subcutaneous administration decreases infusion-related reactions and considerably shortens treatment time [63,64]. Similar challenges may emerge with RAS(ON) inhibitors, including daraxonrasib and elironrasib, which are being evaluated in *KRAS* G12C and non-G12C disease, including in combination with pembrolizumab, with or without chemotherapy (NCT06162221) [65]. In these combinations, immune-related adverse events may complicate the attribution and management of agent-specific toxicities, particularly rash and gastrointestinal events [66]. Supportive care strategies should therefore be incorporated prospectively into their clinical development.

One of the clearest examples of sustained disease control with targeted therapy is represented by lorlatinib, a third-generation inhibitor recommended for patients with *ALK*-rearranged NSCLC and considered in selected patients with *ROS1*-fusion disease. In the 7-year update of the phase III CROWN trial, median PFS with first-line lorlatinib remained unreached, with 55% of patients progression-free at 7 years, whereas OS follow-up remains ongoing. This prolonged exposure is accompanied by clinically relevant toxicities, including grade ≥2 weight gain in 36.6% of patients and cognitive effects in 28% at any grade. Hyperlipidemia and peripheral edema frequently emerge earlier during treatment but may require long-term monitoring and management [67,68].

In this context, beyond preventive and supportive strategies (such as prophylactic dermatologic regimens or the use of statins and other lipid-lowering agents for lorlatinib-associated hyperlipidemia), temporary treatment interruptions or dose reduction may facilitate the resolution or mitigation of persistent adverse events while also reducing the risk of their recurrence [69,70].

For patients without actionable oncogenic drivers, ICIs, alone or in combination with chemotherapy according to PD-L1 expression and histology, represent the standard treatment [71]. In patients achieving prolonged disease control, treatment duration has itself become a survivorship issue. Registrational trials adopted different strategies, ranging from fixed durations, 35 cycles for pembrolizumab in KEYNOTE-024 and 108 weeks for cemiplimab in EMPOWER-Lung 1, to treatment continued until disease progression or unacceptable toxicity, as in CheckMate-026 and IMpower110 [72,73]. Durable benefit can extend for several years. In a highly selected retrospective cohort of patients with metastatic NSCLC who completed at least 2 years of ICI therapy, median time to progression and median OS from ICI initiation were 6.9 and 9.5 years, respectively [74]. Observational studies suggest that treatment discontinuation after 2 years may be a reasonable option for selected patients with ongoing disease control, although randomized evidence is lacking and the decision should remain individualized [75,76,77].

Prolonged clinical benefit may still be accompanied by immune-related toxicity. In a retrospective cohort of patients receiving ICIs for more than 2 years, 50% developed a late immune-related adverse event, and 39% of these patients had experienced no earlier immune-related toxicity [78]. Long-term follow-up should therefore include surveillance for new or persistent adverse events, even in clinically stable patients.

Other treatment modalities, including ADCs, bispecific antibodies, T-cell engagers, and vaccine-based strategies, are likely to further increase the number of patients living for prolonged periods with advanced lung cancer [79,80,81,82]. Their chronic and delayed toxicity profiles remain incompletely characterized, creating additional needs for long-term monitoring and supportive care aimed at preserving quality of life.

### 3.5. Survivorship in Limited- and Extensive-Stage Small-Cell Lung Cancer

In recent years, new therapeutic options have also emerged for patients with small-cell lung cancer (SCLC). In extensive-stage disease, adding atezolizumab or durvalumab to platinum–etoposide established new first-line standards, albeit with modest median OS gains [83,84]. More recently, maintenance lurbinectedin plus atezolizumab improved survival over atezolizumab alone (IMforte), while the DLL3-targeted T-cell engager tarlatamab improved median OS over standard chemotherapy in relapsed disease [81,85]. These strategies may extend survival in extensive-stage SCLC but introduce specific supportive-care needs related to hematologic toxicity, immune-related adverse events, cytokine release syndrome, and neurologic toxicity.

In limited-stage SCLC, surgery within a multimodal approach is an option only for the small minority with very early cT1–2N0 disease, whereas concurrent chemoradiotherapy remains the main curative-intent strategy for most patients. In the ADRIATIC trial, consolidation durvalumab increased median OS, potentially expanding the population requiring long-term surveillance and management of pulmonary, esophageal, cardiac, neurocognitive, and immune-related toxicities [86,87]. Prophylactic cranial irradiation may be offered to patients responding to chemoradiotherapy to reduce the risk of brain metastases, but its neurocognitive long-term sequelae warrant careful patient selection and consideration of hippocampal-avoidance techniques [86,88].

Despite these advances, SCLC is associated with frequent relapse and substantial symptom burden; early integration of palliative care should accompany active treatment, particularly in advanced disease and in patients with uncontrolled symptoms.

## 4. Cross-Cutting Dimensions of Lung Cancer Survivorship

Beyond stage- and treatment-specific toxicities, patients with lung cancer may experience several physical, psychological, social, and financial challenges regardless of disease stage or treatment setting. This section examines the main issues relevant to survivorship care.

### 4.1. Tobacco Cessation as a Component of Survivorship Care

Smoking cessation is a key component of lung cancer prevention and survivorship care. Quitting smoking has been associated with improved survival, including among patients with advanced-stage disease, and may also reduce the burden of smoking-related cardiovascular and respiratory comorbidities [89].

Smoking cessation programs should be structured according to current World Health Organization (WHO) guideline recommendations combining behavioral counseling, psychological support, and pharmacologic treatment, delivered by trained professionals and adapted to patient preferences and local resources [90]. Pharmacologic support represents an essential component of treatment and may include nicotine replacement therapy, bupropion, varenicline, and cytisine, depending on availability and regulatory approval across different countries (Table 2).

Despite its clinical relevance, smoking cessation remains inadequately implemented and quit rates among patients with lung cancer remain suboptimal [91]. Further efforts are therefore needed to move beyond the current paradigm, with greater emphasis on systematic referral, broader implementation of pharmacologic strategies, and integration of novel digital tools [92]. Tobacco-related stigma, including after a cancer diagnosis, remains an important barrier to accessing smoking cessation services, together with logistical challenges and the limited availability of structured institutional policies supporting these interventions [93].

Finally, cessation initiatives remain poorly defined for users of heated tobacco products and e-cigarettes. Although nicotine-containing e-cigarettes may support smoking cessation, evidence regarding their long-term safety and their specific role in lung cancer survivorship remains limited, while heated tobacco products should not be regarded as established cessation tools [94,95].

### 4.2. Integration of Palliative Care Throughout Survivorship

The integration of palliative care into routine oncology practice has been endorsed by international guidelines since 2012 [96]. The evolving therapeutic landscape and the prolonged disease control achieved with contemporary anticancer treatments have raised questions regarding the optimal timing, intensity, and scope of palliative care interventions. In particular, the persistent, cumulative, or recurrent toxicities associated with TKIs and ICIs may require prolonged symptom management and supportive care. Accordingly, palliative care may have an expanding role in addressing treatment-related symptoms and their functional and quality-of-life consequences, building on its established expertise in symptom assessment and control. Early integrated palliative care, also known as simultaneous care, is delivered alongside active anticancer treatment and tailored to patients’ needs throughout the disease course, rather than being reserved for the end of life [97].

However, this model has not been consistently implemented across clinical settings and healthcare systems. Many patients still lack access to palliative care or are referred only late in the disease course, largely because of limited specialist resources [98]. These constraints have prompted the development of more pragmatic and resource-efficient models of care.

One example is a stepped-care approach in which all patients receive an initial palliative care consultation, while subsequent visits are scheduled at clinically relevant transition points, such as a change in anticancer treatment or hospitalization. Quality of life is monitored using the Functional Assessment of Cancer Therapy–Lung questionnaire, with clinically meaningful deterioration prompting escalation to regular palliative care visits. In a randomized noninferiority trial, stepped care was noninferior to standard early palliative care in terms of quality of life while requiring fewer specialist consultations, supporting its potential as a more scalable and resource-efficient model. Its implementation, however, requires reliable tracking of treatment changes, hospitalizations, and patient-reported outcomes, potentially through integrated electronic tools [99].

Other models seek to adapt the timing and scope of palliative care to specific patient populations and clinical pathways. The POISE trial, for example, is evaluating a blended palliative care and survivorship intervention tailored to patients with metastatic oncogene-driven NSCLC [100], whereas the PACO randomized trial assessed the systematic integration of palliative care from diagnosis in patients with previously untreated metastatic NSCLC [101]. Specialist palliative care may also be incorporated directly into rapid diagnostic pathways. In a Canadian academic center, the inclusion of palliative care specialists within a Lung Diagnostic Assessment Program improved both access to and timeliness of assessment among patients with newly diagnosed stage IV lung cancer [102].

Regardless of the model adopted, timely integration of palliative and end-of-life care remains essential to support hospice referral and ensure that treatment decisions are aligned with patients’ goals and preferences [103,104,105]. However, discussions regarding prognosis and end-of-life preferences are often delayed, potentially contributing to non-beneficial treatment near death, poorer quality of life, and higher healthcare costs [106,107,108,109,110].

Automated referral systems, telemedicine, and digital tools for remote symptom monitoring may facilitate earlier access to palliative care and reduce geographical and logistical barriers [111,112,113,114]. These approaches may also help address persistent disparities related to race, sex, and socioeconomic status, although their impact will depend on equitable access to technology, adequate digital literacy support, and the inclusion of underserved populations [115,116,117].

### 4.3. Physical Activity and Rehabilitation

Regular physical activity has been associated with a reduced risk of several malignancies, including lung cancer [118]. Its relevance, however, extends well beyond primary prevention. After a cancer diagnosis, structured exercise interventions may improve physical function, muscle strength, quality of life, cancer-related fatigue, and other treatment-related symptoms during active treatment and throughout survivorship [119,120,121]. These benefits may be particularly relevant for patients with lung cancer, who frequently experience sarcopenia, loss of muscle mass, and progressive functional decline during systemic therapy, while reduced physical activity after diagnosis may also contribute to weight gain and unfavorable changes in body composition [122]. Adapted exercise programs have also demonstrated feasibility and potential clinical benefit in patients with advanced disease, including those receiving palliative care [123,124]. In addition, observational studies have associated higher levels of physical activity before and after diagnosis with improved survival, including among lung cancer survivors [125,126].

Although lung cancer-specific evidence remains limited and warrants further investigation, these findings support moving beyond generic advice to remain active toward individualized exercise assessment, prescription, and referral based on functional status, cardiopulmonary reserve, treatment-related toxicities, comorbidities, and patient preferences [127]. In support of this approach, a Korean nationwide cohort study of 23,257 patients with lung cancer found that both pre-diagnosis and post-diagnosis physical activity were associated with lower all-cause and lung cancer-specific mortality [125].

Despite its endorsement by major oncology and exercise-oncology guidelines and consensus statements [128,129,130], physical activity remains inconsistently integrated into routine oncology and survivorship care. Its implementation is hindered by multilevel barriers, including the limited availability of structured programs and qualified professionals, the absence of standardized assessment and referral pathways, geographic constraints, and financial considerations [131]. This implementation gap contrasts with patients’ expressed interest in receiving structured exercise support and their preference for physical activity to be proactively discussed by the oncology team [132,133]. Further efforts should focus not only on promoting physical activity, but also on establishing accessible, individualized, and sustainable pathways for exercise assessment, prescription, and referral throughout the lung cancer care continuum.

### 4.4. Nutritional Assessment and Support

Nutritional risk should be screened from the time of cancer diagnosis and reassessed regularly throughout treatment and follow-up. Patients identified as being at nutritional risk should undergo a comprehensive nutritional assessment and receive individualized support [128,134]. Dietary impairment may develop at any point during the prolonged course of cancer care and therefore remains highly relevant within survivorship programs, in which substantial unmet nutritional needs have already been identified [135]. Nutritional deficiencies may result from treatment-related adverse events, including mucositis, persistent diarrhea and associated malabsorption, and endocrine dysfunction, as well as from disease-related symptoms such as fatigue, dyspnea, and early satiety. In these populations, particular attention should be paid to cancer cachexia, a multifactorial syndrome characterized by the progressive loss of skeletal muscle mass, with or without loss of fat mass, driven by a combination of reduced nutritional intake, systemic inflammation, and metabolic alterations. Unlike malnutrition, cachexia cannot be fully reversed by conventional nutritional support and is associated with impaired physical function, reduced treatment tolerance, and poorer clinical outcomes [136,137,138].

Longitudinal dietary assessment is therefore essential, given the potential for delayed treatment-related toxicities, including late-onset immune-related adverse events [139].

Conversely, some systemic therapies may lead to progressive and persistent weight gain. This is relevant for lorlatinib, for which grade ≥2 weight gain has been reported in 36.6% of treated patients [67], as well as for entrectinib (7.3%, grade ≥3) [140,141]. For these patients, the promotion of adapted physical activity and a balanced diet represent essential components of supportive and survivorship care [142,143].

Despite the recognized importance of these interventions and their perceived value among patients and survivors [144,145], nutritional counseling and support remain inconsistently available because of limited awareness, insufficient dedicated services, and inadequate resources. Greater investment is therefore needed to ensure their systematic and equitable integration into routine cancer care and survivorship pathways [146,147].

### 4.5. Psychological and Psychosocial Support

Psycho-oncological assessment and support should be integrated from diagnosis onward and reconsidered at clinically relevant intervals and major disease transitions. This is important in lung cancer, as patients and their partners frequently experience anxiety, depression, disease-related distress, unmet psychological needs, and impaired health-related quality of life [148,149,150,151].

Among long-term survivors and patients experiencing prolonged disease control, fear of recurrence or progression may be pronounced around radiologic surveillance, a phenomenon commonly referred to as “scanxiety.” Addressing this concern represents a specific component of survivorship care, together with interventions aimed at helping patients restore daily routines, social roles, and a sense of normalcy after diagnosis and treatment [152,153].

Sleep disturbances are also common among patients with lung cancer and may persist during treatment and survivorship, often occurring alongside fatigue, pain, respiratory symptoms, anxiety, and depression [154]. Their etiology is multifactorial and may reflect cancer-related symptoms, treatment effects, psychological distress, and maladaptive sleep behaviors. Routine assessment may help identify clinically relevant insomnia, which is frequently overlooked in oncology practice. Cognitive behavioral therapy for insomnia (CBT-I) is the recommended first-line intervention and may also improve fatigue and psychological symptoms, although most evidence derives from mixed cancer populations rather than lung cancer-specific studies [155,156]. Brief or digitally delivered behavioral interventions may represent more accessible alternatives when specialist-delivered CBT-I is unavailable.

Current guidelines recommend screening for depression and anxiety at diagnosis, during treatment and follow-up, and at major transitions such as recurrence or disease progression. Management should follow a stepped-care approach, with psychological, psychosocial, and pharmacologic interventions tailored to symptom severity, patient characteristics, and available resources [157].

Digital health interventions may improve the accessibility and scalability of psycho-oncological support. An umbrella review of 78 systematic reviews reported generally favorable effects of web-, app-, and other digitally delivered interventions on depression, anxiety, quality of life, and other psychosocial outcomes, although substantial methodological heterogeneity reduces the certainty of the evidence and warrants further research [158].

### 4.6. Sexual Health and Dysfunction

Sexual health is frequently impaired following a cancer diagnosis and the initiation of treatment, with at least 40% of patients reporting difficulties that may persist over time as a consequence of the disease, treatment-related effects, or both [159,160,161,162]. Sexual dysfunction has a complex biopsychosocial basis and may reflect hormonal, vascular, neurologic, and mucocutaneous alterations, as well as systemic symptoms such as chronic fatigue, anxiety, depression, and changes in body image [163,164,165]. Sexual health concerns have also been documented across thoracic malignancies, disease stages, and treatment modalities and they may also affect intimate partners, with potential consequences for communication, emotional closeness, and couple dynamics [165,166,167,168,169].

In lung cancer, the available evidence consistently indicates a substantial burden, although prevalence estimates vary according to the population and assessment method. As an example, in the SHAWL study, approximately 77% of women reported little or no interest in sexual activity [170] and in the LUDICAS cohort, 63% of 448 patients reported sexual dysfunction after treatment initiation [168], whereas women with lung cancer enrolled in the CLARIFY project had a markedly increased risk of severe arousal dysfunction [171]. Nevertheless, compared with other oncologic settings, evidence specifically addressing sexual health in lung cancer survivorship is limited, underscoring the need for further disease-specific research [170].

Despite its prevalence and potential effect on quality of life, sexual dysfunction is underrecognized in routine cancer care. Relevant barriers include competing clinical priorities, reluctance or embarrassment among both patients and healthcare professionals, inadequate consultation time, and insufficient staff training [172]. Addressing these barriers requires structured organizational and communication strategies [164,173]. Proposed measures include dedicated training for healthcare professionals and the development of multidisciplinary pathways involving oncologists, gynecologists, urologists, psychiatrists, psychologists or sexologists, oncology nurses, and, when appropriate, primary care physicians. Nursing staff may have an important role in screening for sexual health concerns and initiating discussions with patients [163].

Management should begin with the identification and treatment of potentially modifiable contributors to sexual dysfunction [168]. Cancer-related fatigue is among the most relevant factors, and interventions aimed at preventing or mitigating it should be part of the overall management strategy [174]. Lifestyle interventions and physical activity may also improve sexual health outcomes and should be considered early in the care pathway when clinically appropriate [175,176]. Finally, better control of disease-related symptoms such as dyspnea may help address these issues.

In selected patients, pharmacologic therapies may also be considered following appropriate specialist assessment [177,178]. However, the implementation of comprehensive sexual health services may be restricted by financial and organizational barriers, including the costs associated with dedicated interventions and the uneven availability of professionals with the required expertise [163].

### 4.7. Fertility and Its Preservation

The growing recognition of young-onset lung cancer in selected populations, together with the prolonged disease control achieved with contemporary therapies, has made reproductive health an increasingly relevant and still not completely explored component of survivorship. In patients of reproductive potential, the possible effects of anticancer treatment on fertility and the available preservation options need to be discussed before treatment initiation, considering prognosis, treatment urgency, individual preferences, and future reproductive goals [179]. Nevertheless, these discussions are inconsistently incorporated into routine care. In the observational REACT study, approximately half of 473 patients with early-onset cancer did not recall discussing fertility preservation with a healthcare professional before starting treatment [180].

Evidence regarding the reproductive toxicity of contemporary systemic treatments is still limited and is largely derived from preclinical studies or extrapolated from other malignancies. In animal models, exposure to ICIs has been associated with a reduction in ovarian follicular reserve and adverse pregnancy outcomes, including pregnancy loss, stillbirth, and preterm delivery [181,182]. Reproductive function may also be indirectly affected by persistent immune-related endocrinopathies, including hypophysitis and primary or secondary hypogonadism [181,182].

Data on TKIs are similarly sparse, heterogeneous and immature. Available evidence, derived predominantly from older agents used in malignancies other than lung cancer and from experimental models, suggests potential adverse effects on ovarian reserve, oocyte maturation and retrieval, embryonic development, and pregnancy outcomes [183,184].

In men, anticancer treatments may impair spermatogenesis through direct gonadotoxic effects or treatment-related endocrine dysfunction; however, the reproductive effects of contemporary lung cancer therapies remain poorly characterized and are largely extrapolated from other malignancies [185,186,187].

Beyond fertility preservation, pregnancy during active treatment for lung cancer is rare, and evidence guiding oncologic and obstetric management is limited. To address this knowledge gap, the International Pregnancy and Lung Cancer Registry was established to prospectively collect data on treatment exposure and maternal, fetal, and oncologic outcomes. An initial analysis from the registry, including 22 cases, highlighted substantial heterogeneity in treatment selection, treatment deferral, and imaging practices [188]. Overall, lung cancer-specific evidence on gonadal toxicity, fertility preservation, and reproductive outcomes is insufficient, underscoring the need for prospective data collection and the systematic integration of reproductive counseling into the care of younger patients.

### 4.8. Older Adults, Frailty, and Survivorship

Population aging, especially in high-income countries, has resulted in a growing proportion of older adults among individuals diagnosed with lung cancer. The median age at diagnosis is approximately 71 years, and more than 70% of cases occur in adults older than 65 years [189,190].

In this population, which is frequently characterized by comorbidities and polypharmacy, international guidelines recommend geriatric assessment and have incorporated it into diagnostic and therapeutic algorithms [191]. Frailty should not be equated with chronological age, comorbidity, or disability, although these conditions frequently overlap [192]. Its evaluation therefore requires a comprehensive geriatric assessment encompassing functional status, mobility, cognition, nutrition, comorbidities, and polypharmacy [193]. In this regard, the prognostic relevance of frailty was confirmed in a population-based study of 1778 lung cancer survivors, in which frail individuals had more than twice the risk of mortality compared with non-frail survivors [194].

Beyond its prognostic value for survival, geriatric vulnerability is also a determinant of functional trajectory. Screening tools such as the G8 have been shown to predict functional decline in older patients with lung cancer, while longitudinal studies, including the FITNESS study, are evaluating how these measures evolve over time in relation to treatment toxicity and disability [195]. This is relevant in the survivorship setting, as many older adults prioritize the preservation of functional independence over prolonged survival alone, making functional decline a key patient-centered outcome irrespective of OS.

A comprehensive assessment should also include the patient’s social and home environment, with the aim of preventing falls, social isolation, and inadequate support. These dimensions are essential not only for treatment delivery but also for long-term care planning and survivorship outcomes.

The structured implementation of geriatric oncology pathways is becoming progressively important as therapeutic advances prolong survival and treatment exposure in older patients. The long-term tolerability of newer anticancer treatments in older adults remains incompletely characterized, particularly in the presence of frailty, cognitive vulnerability, comorbidities, and polypharmacy [196]. Chronic toxicities, including neurocognitive effects associated with selected TKIs and persistent or late-onset immune-related adverse events, may be especially challenging to recognize and manage in this population [197]. This further supports the need for longitudinal geriatric assessment and multidisciplinary care. However, organizational, workforce, and financial barriers continue to limit the widespread implementation of dedicated geriatric oncology programs, with potential consequences for both patients and caregivers.

### 4.9. Vaccination and Infection Prevention

Patients with cancer may have impaired immune function because of the malignancy itself or the treatments they receive. This may increase their susceptibility to infections, which can adversely affect the course of the underlying cancer, interfere with treatment delivery, and contribute to additional morbidity and mortality [198,199].

From a survivorship perspective, infection prevention is particularly important because the immunologic effects of anticancer treatments may persist over time. Vaccination status should therefore be assessed at diagnosis, and recommended vaccines should ideally be administered before the initiation of anticancer treatment whenever clinically feasible. When vaccination has not been completed beforehand, appropriate non-live vaccines may also be administered during treatment, depending on the treatment regimen, the patient’s immune status, and the expected immunogenicity of the vaccine. Vaccination requirements should also be regularly reassessed during long-term follow-up [200].

This approach is relevant for patients treated with local therapies, including lung surgery and thoracic radiotherapy. Although these treatments do not necessarily result in systemic immunosuppression, they may reduce pulmonary reserve and increase vulnerability to clinically significant respiratory complications. This is especially relevant in patients with lung cancer, who are frequently older and may have a history of smoking, chronic obstructive pulmonary disease, cardiovascular disease, or other comorbidities that further increase the clinical impact of respiratory infections [199,201].

Several international guidelines therefore recommend a comprehensive assessment of each patient’s vaccination history and the administration of appropriate vaccines based on age, previous treatments, immune status, comorbidities, and individual risk factors. A major consideration concerns live attenuated vaccines, which may pose a significant safety risk during periods of severe immunosuppression and are therefore generally contraindicated in this setting.

By contrast, non-live vaccines can generally be administered safely, although their immunogenicity may be reduced during periods of treatment-related immunosuppression [200].

**Figure 3:**
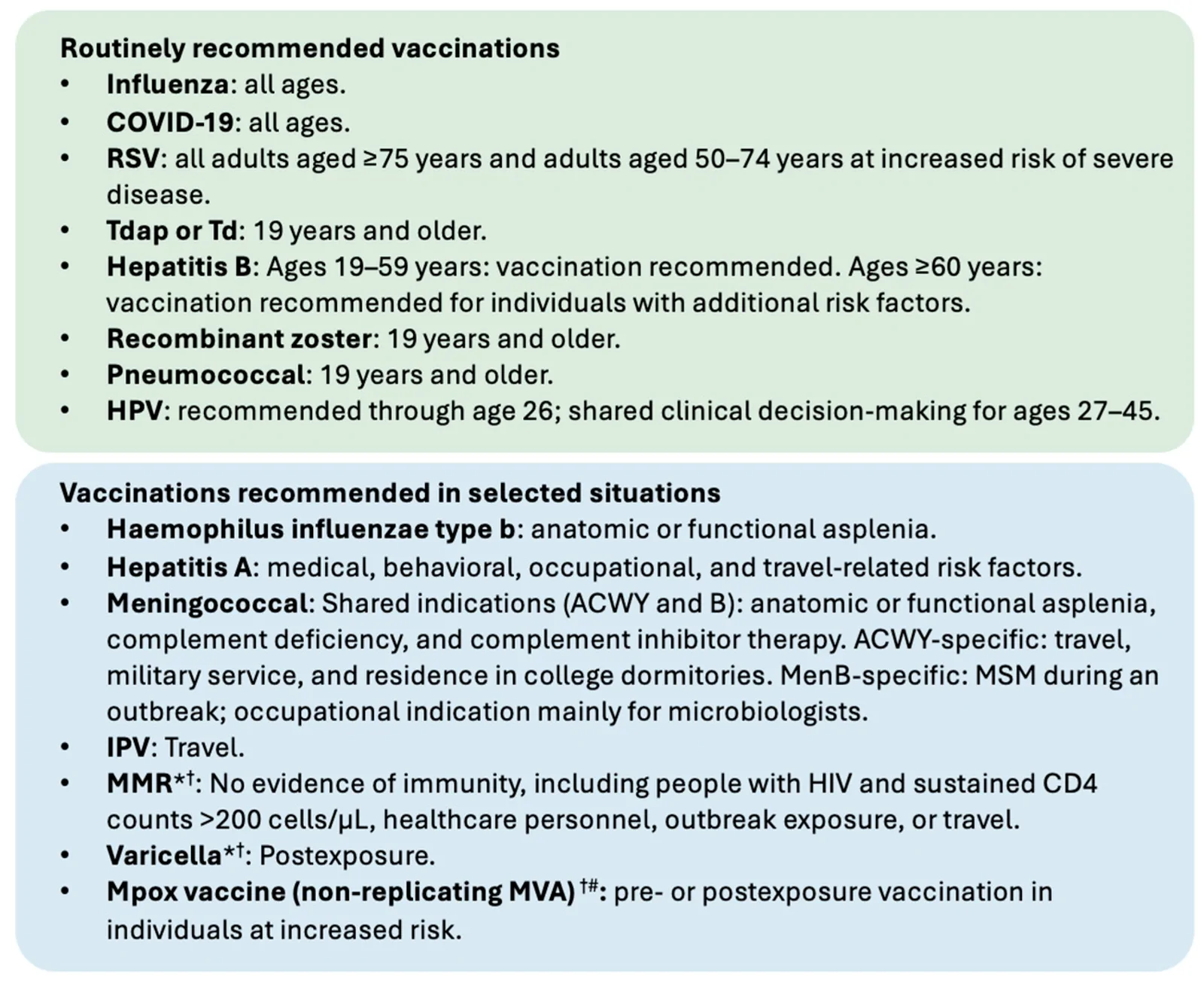
Recommended vaccination strategies for adults with cancer according to current ASCO guidance [200]. RSV, respiratory syncytial virus; Tdap, tetanus, diphtheria, and acellular pertussis vaccine; Td, tetanus and diphtheria; HPV, human papillomavirus; IPV, inactivated poliovirus vaccine; MMR, measles, mumps, and rubella; HIV, human immunodeficiency virus; MVA, modified vaccinia Ankara. * Contraindicated with cancer treatment and other immunocompromising conditions; ^†^ live vaccine; ^#^ Can be safely administered to people with HIV and to those receiving immunosuppressive therapy.

Evidence specifically addressing vaccination strategies in lung cancer survivors is limited; therefore, current recommendations are largely extrapolated from broader guidelines for adults with cancer (Figure 3) [200]. Importantly, recommendations for appropriate and complete vaccination also extend to household members and other close contacts, thereby reducing the risk of preventable infections among vulnerable patients.

### 4.10. Financial Toxicity

Financial toxicity is an increasingly recognized component of survivorship care, including among patients with lung cancer. Its magnitude varies across geographic, socioeconomic, and healthcare contexts, with substantial differences between publicly funded and predominantly private systems [202].

Beyond these structural factors, disease-related symptoms and treatment demands may reduce or interrupt employment, with consequences extending beyond the individual patient to the broader household and family context [203,204].

Several characteristics have been associated with a greater risk of financial toxicity, including age younger than 65 years, low household income, and unemployment. Treatment-related factors may also contribute, including radiotherapy rather than surgery for early-stage disease or receipting systemic therapy. Financial toxicity is also closely associated with unmet supportive care needs and poorer quality of life. In one study, greater overall unmet needs (r = −0.42), as well as physical (r = −0.26), social (r = −0.58), emotional (r = −0.27), and medical needs (r = −0.31), were associated with greater financial burden [205].

Potential mitigation strategies include multidisciplinary financial navigation delivered by financial counselors, social workers, or nurse navigators, with interventions focused on financial education, practical assistance, and support with health insurance enrollment [206].

However, evidence specifically addressing long-term lung cancer survivors is scarce, highlighting an important area for future research. As discussed above, the consequences of financial toxicity may also extend to caregivers, further amplifying the overall burden of the disease and its treatment [207].

### 4.11. Caregiver Burden and Family Dynamics

Cancer care extends beyond patients to include their caregivers, consistent with the NCCS definition, which recognizes family members and loved ones as part of the survivorship experience. Informal caregivers, most commonly partners and adult children, often provide essential practical and emotional support, including assistance with daily activities, symptom monitoring, medication management, transportation, and communication with healthcare professionals. Yet, while patients’ needs are increasingly recognized as central to cancer care, those of caregivers remain underestimated.

Caregivers often report feeling unprepared to manage symptoms and to cope with critical phases of the disease trajectory, including progression [208,209,210]. In these situations, older caregivers may face additional challenges, as they often provide intensive care while managing their own chronic conditions, physical fatigue, and functional limitations [211]. Psychological and spiritual distress are also common; caregivers are at increased risk of depression and insomnia, with negative effects on work productivity and daily activities that may further contribute to social isolation [212,213]. Caregiving responsibilities may also lead to reduced working hours, occupational disruption, and direct and indirect expenses, further increasing the household economic burden of the disease, as discussed in the section on financial toxicity. Caregiver support should also extend into bereavement, with proactive assessment and targeted interventions for those at risk of persistent grief, anxiety, depression, or other mental health difficulties after the patient’s death [214,215].

Despite the clinical relevance of these issues, evidence on interventions for lung cancer caregivers remains limited [216]. Dyadic approaches involving both patients and caregivers may offer a more appropriate framework for supportive care, although data on the effectiveness of specific interventions remain heterogeneous. Moreover, the implications of longer survival for caregiver burden and supportive care needs are insufficiently characterized [149,217].

### 4.12. Patient Advocacy and Survivorship Support

The increasing complexity of lung cancer care may leave patients facing uncertainty regarding treatment selection, expected long-term benefit, and the potential burden of treatment-related toxicities. In this context, peer-to-peer support and patient advocacy organizations can provide important informational, emotional, and psychosocial support throughout the disease trajectory [218].

Lung cancer advocacy developed later than in several other cancer settings, partly because tobacco-related stigma and patient blame have limited public engagement [219,220]. Early initiatives such as the Alliance for Lung Cancer Advocacy, Support and Education, which subsequently evolved into Lung Cancer Alliance and later GO2 for Lung Cancer, contributed to strengthening patient representation.

Several organizations now operate across different geographic and clinical settings. In Europe, Lung Cancer Europe represents a network of national patient organizations, while Women Against Lung Cancer in Europe has developed initiatives addressing molecular profiling, access to clinical trials, and primary and secondary prevention, including the EPROPA program [221]. In the United States, organizations such as GO2 for Lung Cancer and the LUNGevity Foundation have promoted patient support, survivorship initiatives, research advocacy, and more inclusive and pragmatic clinical trial designs. Precision oncology has also encouraged the development of biomarker-specific communities representing patients with actionable alterations (e.g., ALK-Positive, EGFR Resisters, ROS1ders), thereby increasing the visibility of populations that may otherwise remain underrepresented.

The role of advocacy organizations now extends across the entire lung cancer care continuum. Their activities include tobacco prevention and awareness, promotion of lung cancer screening, support during diagnosis and treatment, and assistance with issues that emerge during long-term survivorship. Their involvement is also becoming progressively relevant in early-stage disease, as neoadjuvant, perioperative, and adjuvant strategies may increase treatment complexity and perceived burden [222]. Within survivorship care, advocacy organizations may address quality of life, fear of recurrence, treatment-related toxicities, financial hardship, patient navigation, and access to reliable information [205,223,224]. They may also involve patients as research partners in protocol development, clinical trial design, and regulatory discussions.

Despite this progress, evidence formally evaluating the impact of advocacy organizations on survivorship outcomes remains scarce. Access to their services is also uneven and may be restricted by geographic isolation, socioeconomic disadvantages, language barriers, cultural differences, and racial or ethnic disparities [225]. More systematic evaluation and broader, equitable access are therefore needed to define and strengthen their contribution to comprehensive lung cancer survivorship care.

## 5. Discussion and Future Directions

Cancer survivorship has become a major area of interest, particularly for highly prevalent malignancies associated with favorable long-term survival, such as breast and prostate cancer [226]. Attention is now increasingly directed toward lung cancer care, as more patients are diagnosed with early-stage disease or achieve prolonged disease control with TKIs or ICIs and consequently live substantially longer than before [7,227].

Despite this rising relevance, an important question remains: are healthcare systems truly prepared for such a substantial shift in the traditional model of lung cancer care? In this regard, several survivorship models have been proposed, and a growing number of studies are now focusing on the needs of this expanding population [7,205]. The available evidence, however, is limited and largely centered on patients with early-stage disease treated with curative intent, whereas survivorship in locally advanced and metastatic disease has received considerably less attention [228].

**Figure 4:**
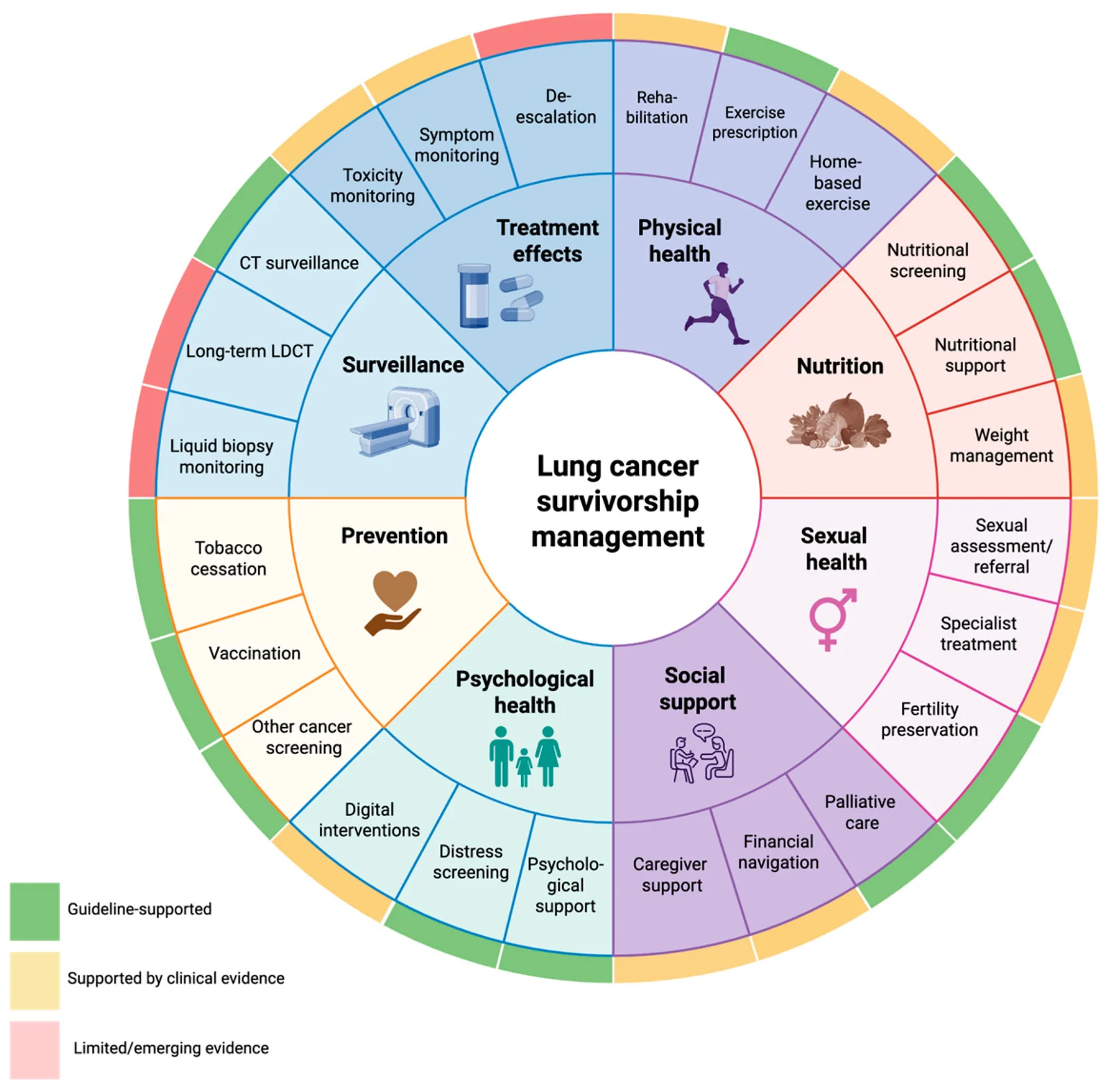
Main domains of lung cancer survivorship care, corresponding management strategies, and current status of supporting evidence. LDCT, low-dose computed tomography.

In this context, Figure 4 provides a clinically oriented synthesis of the main domains of lung cancer survivorship care, linking each domain to relevant management strategies and the current status of supporting evidence.

Survivorship care in lung cancer also encompasses interventions with no equivalent in most other malignancies, notably sustained smoking cessation support, which continues to influence outcomes well beyond the initial treatment phase, and long-term surveillance for second primary lung cancers, to which these patients remain susceptible [229,230].

Current models range from hospital-based programs, often supported by dedicated nursing staff during routine follow-up [231,232] to more decentralized approaches that deliver selected interventions, such as physical activity programs, remotely or directly in patients’ homes [233]. A hybrid model combining specialized hospital-based services with telemedicine and remote supportive care may offer a feasible strategy to ensure continuity of care while improving access for geographically isolated, socioeconomically disadvantaged, and other underserved populations [234].

Within this framework, digital tools, including wearable devices and mobile applications, may support longitudinal survivorship care through the remote collection of patient-reported symptoms, functional measures, health-related quality of life, and supportive care needs, potentially facilitating the earlier identification of unmet needs [235].

These technologies could therefore contribute to more proactive and personalized models of care. Their effectiveness, however, will depend on equitable access, adequate digital literacy, interoperability with existing healthcare systems, and the availability of clinical teams able to review and act on the information collected [236].

Beyond their role in monitoring symptoms and supportive care needs, emerging technologies may also influence oncologic surveillance and its psychological impact on patients. Fear of recurrence or progression is a major survivorship concern among patients treated with curative intent and those experiencing prolonged disease control, and may become especially pronounced around radiologic assessments [152,153].

In the same context, emerging approaches, including liquid biopsy and circulating tumor DNA analysis, may complement imaging by enabling earlier detection of molecular residual disease or recurrence, although their role in routine lung cancer surveillance and survivorship remains to be established [237,238]. Furthermore, while these tools could support more personalized follow-up, they may also introduce new psychological, clinical, and organizational challenges, particularly when uncertain or clinically indeterminate findings must be interpreted and communicated. Accordingly, these approaches should currently be considered exploratory.

Given the heterogeneity of patients living with and beyond lung cancer, the development of structured and comprehensive assessment platforms is urgently needed. Such models should integrate stage and treatment-specific toxicities with physical, psychological, social, nutritional, functional, financial, and caregiver-related needs. They should also be sufficiently flexible to accommodate patients who have completed curative-intent treatment, those living with chronic or persistent disease, and those whose goals of care evolve over time [8].

Alongside these technological developments, the human and organizational infrastructure of survivorship care remains equally decisive. Dedicated personnel, such as survivorship nurses and patient navigators, can coordinate follow-up, identify unmet needs, and guide patients through progressively complex care pathways, while structured training may help oncology teams recognize and address survivorship issues that extend beyond disease control [100,231,239]. In parallel, the early integration of palliative care should be tailored to disease stage, symptom burden, prognosis, patient needs, and available resources, rather than implying universal longitudinal specialist palliative-care follow-up [240]. Ultimately, the value of digital and remote tools will depend on their embedding within adequately resourced multidisciplinary teams, without which technological solutions risk generating information that cannot be acted upon [236].

This narrative review provides a broad overview of the principal stage-specific and cross-cutting dimensions of lung cancer survivorship. However, several limitations should be acknowledged. First, because of its narrative design, the literature was not assessed through a formal systematic review process, and study selection may therefore have been influenced by author judgment. Second, evidence supporting several survivorship interventions is partial and is frequently derived from observational studies, small cohorts, or heterogeneous patient populations. Third, despite the broad scope of the review, some domains relevant to patients may not have been appropriately addressed. Fourth, the applicability of some recommendations may also vary across countries according to healthcare organization, resource availability, socioeconomic context, and local policies. Future research should aim to better define survivorship outcomes across disease stages, particularly in advanced disease, and to establish risk-adapted strategies for monitoring chronic and delayed treatment-related toxicities. Patient and caregiver-centered models, including digitally supported interventions, should also be prospectively evaluated in terms of their cost-effectiveness, scalability, and equitable implementation, while the identification of core patient-reported outcomes may facilitate more standardized survivorship pathways.

## 6. Conclusions

Lung cancer survivorship is an expanding and heterogeneous field that extends across all disease stages. Comprehensive care should move beyond oncologic surveillance to address treatment-related toxicities and patients’ physical, psychological, functional, social, and financial needs with flexible, multidisciplinary, and risk-adapted models, supported by digital tools and early palliative care integration. Finally, prospective studies should define standardized survivorship outcomes and assess the effectiveness and equitable implementation of sustainable models of care.

## Figures and Tables

**Table 1:** Most commonly used targeted and biomarker-directed therapies in NSCLC: early-onset and potentially persistent adverse events. These categories are not mutually exclusive, as early-onset adverse events may persist during prolonged treatment, and some toxicities may emerge after variable treatment durations. CNS, central nervous system; CPK, creatine phosphokinase; GI, gastrointestinal; ILD, interstitial lung disease; LVEF, left ventricular ejection fraction.

Molecular Alterations	Treatment or Combination	Common Early Onset Adverse Events	Potentially Persistent, Cumulative, or Late Adverse Events
Common *EGFR* mutations	Osimertinib ± platinum–pemetrexed	Diarrhea, rash, mucositis, fatigue, liver enzyme increase; myelosuppression with chemotherapy	Skin and nail toxicity, persistent diarrhea, fatigue; renal impairment for chemotherapy
*EGFR* ex.20 insertions, common *EGFR* mutations	Amivantamab-based combinations	Administration-related reactions, acneiform rash, diarrhea, mucositis; myelosuppression with chemotherapy	Skin and nail toxicity, edema, hypoalbuminemia, fatigue
*KRAS* p.G12C	Sotorasib, adagrasib	Diarrhea, fatigue, musculoskeletal pain, liver enzyme increase	Diarrhea, fatigue, liver enzyme increase; renal impairment with adagrasib
*HER2*	Trastuzumab-deruxtecan	Diarrhea, fatigue, myelosuppression	Fatigue, alopecia, ILD/pneumonitis, decreased LVEF
	Sevabertinib, zongertinib	Diarrhea, rash, stomatitis, nausea, liver enzyme increase	Persistent GI and skin toxicity; ocular or pancreatic toxicity
*MET*	Tepotinib, capmatinib	GI toxicity, fatigue, rash, creatinine increase	Peripheral edema, hypoalbuminemia, renal impairment, pleural effusion
	Telisotuzumab -vedotin	Administration-related reactions, fatigue, GI and ocular toxicities	Neuropathy, edema, keratitis, weight loss
*RET*	Selpercatinib, pralsetinib	GI toxicity, fatigue, hypertension, edema, liver enzyme increase; myelosuppression with pralsetinib	Chronic hypertension, edema, fatigue, GI and liver abnormalities; persistent myelosuppression with pralsetinib
*ALK*	Alectinib, brigatinib	GI toxicity, fatigue, myalgia, edema, liver abnormalities	Persistent myalgia, edema, fatigue; hypertension and metabolic or pancreatic abnormalities with brigatinib
	Lorlatinib	Dyslipidemia, edema, diarrhea, fatigue, cognitive/mood effects, liver enzymes increase	Dyslipidemia, weight gain, peripheral neuropathy, chronic edema, cognitive/mood effects
*ROS1*	Crizotinib, entrectinib, repotrectinib, taletrectinib	Dizziness, ataxia, GI toxicity, fatigue, CPK/liver enzyme increase	Neurologic and GI toxicity, neuropathy and myalgia; hyperuricemia with taletrectinib
*NTRK*	Entrectinib, larotrectinib	Dizziness, GI toxicity, fatigue, edema, liver enzyme increase	CNS effects, weight gain, edema, neuropathy, anemia
*BRAF* V600E	Dabrafenib–trametinib, encorafenib–binimetinib	Pyrexia, GI toxicity, rash, fatigue, musculoskeletal symptoms	Pyrexia, fatigue, musculoskeletal pain, hypertension, ocular toxicity, decreased LVEF
*NRG1*	Zenocutuzumab	Administration-related reactions, GI toxicity, fatigue, dyspnea, rash	Persistent fatigue and GI symptoms, edema, dyspnea, musculoskeletal pain, decreased LVEF

**Table 2:** Pharmacologic strategies for tobacco cessation according to WHO [90].

Treatment	Modality	Mechanisms of Action	Side Effects
Nicotine replacement therapy	Long-acting transdermal patch; short-acting gum, lozenge, mouth spray, nasal spray, or inhaler	Nicotine receptor stimulation, reducing cravings and withdrawal	Skin irritation and sleep disturbance with patches; mouth or throat irritation, hiccups, dyspepsia, and nausea with oral formulations
Bupropion	Oral sustained-release tablets	Dopamine and norepinephrine reuptake inhibition	Insomnia, dry mouth, headache, and nausea; rare dose-related seizures
Varenicline	Oral tablets	Partial agonism at α4β2 nicotinic receptors	Nausea, insomnia, abnormal or vivid dreams, and headache
Cytisine	Oral tablets	Partial agonism at α4β2 nicotinic receptors	Nausea, dry mouth, abdominal discomfort, sleep disturbance, headache, and dizziness

## Data Availability

No new datasets were generated in this study. The complete search strategies and the records retrieved through the literature searches are available from the corresponding author upon reasonable request.

## Abbreviations

The following abbreviations are used in this manuscript:

ADC	Antibody–drug conjugate
ASCO	American Society of Clinical Oncology
CBT-I	Cognitive behavioral therapy for insomnia
CNS	Central nervous system
CPK	Creatine phosphokinase
CT	Computed tomography
GI	Gastrointestinal
HIV	Human immunodeficiency virus
HPV	Human papillomavirus
ICI	Immune checkpoint inhibitor
ILD	Interstitial lung disease
IPV	Inactivated poliovirus vaccine
LDCT	Low-dose computed tomography
LVEF	Left ventricular ejection fraction
MMR	Measles, mumps, and rubella vaccine
MVA	Modified vaccinia Ankara
NCCS	National Coalition for Cancer Survivorship
NSCLC	Non-small-cell lung cancer
OS	Overall survival
PD-1	Programmed cell death protein 1
PD-L1	Programmed death-ligand 1
PFS	Progression-free survival
RSV	Respiratory syncytial virus
SBRT	Stereotactic body radiation therapy
SCLC	Small-cell lung cancer
Td	Tetanus and diphtheria vaccine
Tdap	Tetanus, diphtheria, and acellular pertussis vaccine
TKI	Tyrosine kinase inhibitor
WHO	World Health Organization

## References

[1] Meyer M.L., Fitzgerald B.G., Paz-Ares L., Cappuzzo F., Janne P.A., Peters S., Hirsch F.R. (2024). New promises and challenges in the treatment of advanced non-small-cell lung cancer. Lancet.

[2] Huang Q., Li Y., Huang Y., Wu J., Bao W., Xue C., Li X., Dong S., Dong Z., Hu S. (2025). Advances in molecular pathology and therapy of non-small cell lung cancer. Signal Transduct. Target. Ther..

[3] Siegel R.L., Kratzer T.B., Wagle N.S., Sung H., Jemal A. (2026). Cancer statistics, 2026. CA Cancer J. Clin..

[4] Reck M., Rodriguez-Abreu D., Robinson A.G., Hui R., Csoszi T., Fulop A., Gottfried M., Peled N., Tafreshi A., Cuffe S. (2021). Five-Year Outcomes with Pembrolizumab Versus Chemotherapy for Metastatic Non-Small-Cell Lung Cancer with PD-L1 Tumor Proportion Score ≥ 50%. J. Clin. Oncol..

[5] Shaw A.T., Solomon B.J., Felip E., Bauer T.M., Liu G., Goto Y., Wu Y.L., Soo R.A., Mazieres J., Kim D.W. (2026). Lorlatinib versus crizotinib as first-line treatment for advanced ALK-positive non-small-cell lung cancer: 7-year update from the phase III CROWN study. Ann. Oncol..

[6] Mollica M.A., Doose M., Reed C., Tonorezos E. (2025). Defining concepts in cancer survivorship. Cancer.

[7] Presley C.J., Dalal N., Davenport A.P., Gounden A., Ramchandran K., Tonorezos E. (2024). Survivorship in Advanced Lung Cancer: Understanding a New Landscape and Opportunities. Am. Soc. Clin. Oncol. Educ. Book.

[8] Hart N.H., Nekhlyudov L., Smith T.J., Yee J., Fitch M.I., Crawford G.B., Koczwara B., Ashbury F.D., Lustberg M.B., Mollica M. (2024). Survivorship Care for People Affected by Advanced or Metastatic Cancer: MASCC-ASCO Standards and Practice Recommendations. JCO Oncol. Pract..

[9] IASLC Language Guide. https://www.iaslc.org/IASLCLanguageGuide.

[10] Almatrafi A., Thomas O., Callister M., Gabe R., Beeken R.J., Neal R. (2023). The prevalence of comorbidity in the lung cancer screening population: A systematic review and meta-analysis. J. Med. Screen..

[11] Brunelli A., Hardavella G., Huber R.M., Berghmans T., Frille A., Rodriguez M., Tietzova I., Depypere L., Asteggiano R., Batchelor T. (2025). European Respiratory Society and European Society of Thoracic Surgeons clinical practice guideline on fitness for curative intent treatment of lung cancer. Eur. Respir. J..

[12] Oudkerk M., Liu S., Heuvelmans M.A., Walter J.E., Field J.K. (2021). Lung cancer LDCT screening and mortality reduction—Evidence, pitfalls and future perspectives. Nat. Rev. Clin. Oncol..

[13] Revel M.P., Goo J.M., Vliegenthart R., Silva M., Snoeckx A. (2026). Smoking-Related Comorbidities Detected Through Low-Dose CT Imaging Lung Cancer Screening: Current Evidence and Future Directions. Chest.

[14] Aberle D.R., Adams A.M., Berg C.D., Black W.C., Clapp J.D., Fagerstrom R.M., Gareen I.F., Gatsonis C., Marcus P.M., National Lung Screening Trial Research Team (2011). Reduced lung-cancer mortality with low-dose computed tomographic screening. N. Engl. J. Med..

[15] de Koning H.J., van der Aalst C.M., de Jong P.A., Scholten E.T., Nackaerts K., Heuvelmans M.A., Lammers J.J., Weenink C., Yousaf-Khan U., Horeweg N. (2020). Reduced Lung-Cancer Mortality with Volume CT Screening in a Randomized Trial. N. Engl. J. Med..

[16] Zer A., Ahn M.J., Barlesi F., Bubendorf L., De Ruysscher D., Garrido P., Gautschi O., Hendriks L.E., Janne P.A., Kerr K.M. (2025). Early and locally advanced non-small-cell lung cancer: ESMO Clinical Practice Guideline for diagnosis, treatment and follow-up. Ann. Oncol..

[17] Wolf A.M.D., Oeffinger K.C., Shih T.Y., Walter L.C., Church T.R., Fontham E.T.H., Elkin E.B., Etzioni R.D., Guerra C.E., Perkins R.B. (2024). Screening for lung cancer: 2023 guideline update from the American Cancer Society. CA Cancer J. Clin..

[18] Lee R.W., Nair A., Balata H., Graham C., Parylo C., Abell J., Woodall M., Lawrie M., Mouland S., Brain K. (2026). Implementation of the NHS England Lung Cancer Screening Programme over 5 years. Nat. Med..

[19] Jensen S.O., Moore D.A., Surani A.A., Crosbie P.A.J., Rosenfeld N., Rintoul R.C. (2024). Second Primary Lung Cancer—An Emerging Issue in Lung Cancer Survivors. J. Thorac. Oncol..

[20] Rivera M.P., Benefield T., Durham D.D., Lane L.M., Fiscella K., Mohile S., Henderson L.M. (2025). Lung Cancer Screening in Cancer Survivors vs. Those without a History of Cancer. JAMA Netw. Open.

[21] Rampinelli C., De Marco P., Origgi D., Maisonneuve P., Casiraghi M., Veronesi G., Spaggiari L., Bellomi M. (2017). Exposure to low dose computed tomography for lung cancer screening and risk of cancer: Secondary analysis of trial data and risk-benefit analysis. BMJ.

[22] Yang L., Liu H., Han J., Xu S., Zhang G., Wang Q., Du Y., Yang F., Zhao X., Shi G. (2023). Ultra-low-dose CT lung screening with artificial intelligence iterative reconstruction: Evaluation via automatic nodule-detection software. Clin. Radiol..

[23] Jiang B., Li N., Shi X., Zhang S., Li J., de Bock G.H., Vliegenthart R., Xie X. (2022). Deep Learning Reconstruction Shows Better Lung Nodule Detection for Ultra-Low-Dose Chest CT. Radiology.

[24] Sverzellati N., Silva M., Calareso G., Galeone C., Marchiano A., Sestini S., Sozzi G., Pastorino U. (2016). Low-dose computed tomography for lung cancer screening: Comparison of performance between annual and biennial screen. Eur. Radiol..

[25] Pastorino U., Boeri M., Sestini S., Sabia F., Milanese G., Silva M., Suatoni P., Verri C., Cantarutti A., Sverzellati N. (2022). Baseline computed tomography screening and blood microRNA predict lung cancer risk and define adequate intervals in the BioMILD trial. Ann. Oncol..

[26] Zahed H., Feng X., Alcala K., Smith-Byrne K., Moez E., Guida F., Albanes D., Weinstein S.J., Arslan A.A., Cai Q. (2026). Biomarker-Based Eligibility for Lung Cancer Screening: Validation of the Protein-Based INTEGRAL-Risk Model. JAMA.

[27] Corkum M., Hayden J.A., Kephart G., Urquhart R., Schlievert C., Porter G. (2013). Screening for new primary cancers in cancer survivors compared to non-cancer controls: A systematic review and meta-analysis. J. Cancer Surviv..

[28] Felip E., Altorki N., Zhou C., Vallieres E., Martinez-Marti A., Rittmeyer A., Chella A., Reck M., Goloborodko O., Huang M. (2023). Overall survival with adjuvant atezolizumab after chemotherapy in resected stage II-IIIA non-small-cell lung cancer (IMpower010): A randomised, multicentre, open-label, phase III trial. Ann. Oncol..

[29] Tsuboi M., Herbst R.S., John T., Kato T., Majem M., Grohe C., Wang J., Goldman J.W., Lu S., Su W.C. (2023). Overall Survival with Osimertinib in Resected EGFR-Mutated NSCLC. N. Engl. J. Med..

[30] Wu Y.L., Dziadziuszko R., Ahn J.S., Barlesi F., Nishio M., Lee D.H., Lee J.S., Zhong W., Horinouchi H., Mao W. (2024). Alectinib in Resected ALK-Positive Non-Small-Cell Lung Cancer. N. Engl. J. Med..

[31] Forde P.M., Spicer J., Lu S., Provencio M., Mitsudomi T., Awad M.M., Felip E., Broderick S.R., Brahmer J.R., Swanson S.J. (2022). Neoadjuvant Nivolumab plus Chemotherapy in Resectable Lung Cancer. N. Engl. J. Med..

[32] Wakelee H., Liberman M., Kato T., Tsuboi M., Lee S.H., Gao S., Chen K.N., Dooms C., Majem M., Eigendorff E. (2023). Perioperative Pembrolizumab for Early-Stage Non-Small-Cell Lung Cancer. N. Engl. J. Med..

[33] Heymach J.V., Harpole D., Mitsudomi T., Taube J.M., Galffy G., Hochmair M., Winder T., Zukov R., Garbaos G., Gao S. (2023). Perioperative Durvalumab for Resectable Non-Small-Cell Lung Cancer. N. Engl. J. Med..

[34] Pozo C.L., Morgan M.A., Gray J.E. (2014). Survivorship issues for patients with lung cancer. Cancer Control.

[35] Heiden B.T., Eaton D.B., Chang S.H., Yan Y., Schoen M.W., Chen L.S., Smock N., Patel M.R., Kreisel D., Nava R.G. (2022). The Impact of Persistent Smoking After Surgery on Long-term Outcomes After Stage I Non-small Cell Lung Cancer Resection. Chest.

[36] Wang J.W., Gong X.H., Ding N., Chen X.F., Sun L., Tang Z., Yu D.H., Yuan Z.P., Wang X.D., Yu J.M. (2015). The influence of comorbid chronic diseases and physical activity on quality of life in lung cancer survivors. Support. Care Cancer.

[37] Moller A., Sartipy U. (2012). Predictors of postoperative quality of life after surgery for lung cancer. J. Thorac. Oncol..

[38] Menna C., Rendina E.A., D’Andrilli A. (2022). Parenchymal Sparing Surgery for Lung Cancer: Focus on Pulmonary Artery Reconstruction. Cancers.

[39] Bendixen M., Jorgensen O.D., Kronborg C., Andersen C., Licht P.B. (2016). Postoperative pain and quality of life after lobectomy via video-assisted thoracoscopic surgery or anterolateral thoracotomy for early stage lung cancer: A randomised controlled trial. Lancet Oncol..

[40] Travis L.B., Fossa S.D., Sesso H.D., Frisina R.D., Herrmann D.N., Beard C.J., Feldman D.R., Pagliaro L.C., Miller R.C., Vaughn D.J. (2014). Chemotherapy-induced peripheral neurotoxicity and ototoxicity: New paradigms for translational genomics. J. Natl. Cancer Inst..

[41] Ni Y., Lei J., Huang W., Wang J., Guo H., Lv F., Kang S., Lan K., Jiang T. (2023). Systematic review of the perioperative immunotherapy in patients with non-small cell lung cancer: Evidence mapping and synthesis. Front. Oncol..

[42] Fletcher K., Johnson D.B. (2024). Chronic immune-related adverse events arising from immune checkpoint inhibitors: An update. J. Immunother. Cancer.

[43] Antonia S.J., Villegas A., Daniel D., Vicente D., Murakami S., Hui R., Yokoi T., Chiappori A., Lee K.H., de Wit M. (2017). Durvalumab after Chemoradiotherapy in Stage III Non-Small-Cell Lung Cancer. N. Engl. J. Med..

[44] Spigel D.R., Faivre-Finn C., Gray J.E., Vicente D., Planchard D., Paz-Ares L., Vansteenkiste J.F., Garassino M.C., Hui R., Quantin X. (2022). Five-Year Survival Outcomes From the PACIFIC Trial: Durvalumab After Chemoradiotherapy in Stage III Non-Small-Cell Lung Cancer. J. Clin. Oncol..

[45] Lu S., Kato T., Dong X., Ahn M.J., Quang L.V., Soparattanapaisarn N., Inoue T., Wang C.L., Huang M., Yang J.C. (2024). Osimertinib after Chemoradiotherapy in Stage III EGFR-Mutated NSCLC. N. Engl. J. Med..

[46] Steuer C.E., Behera M., Ernani V., Higgins K.A., Saba N.F., Shin D.M., Pakkala S., Pillai R.N., Owonikoko T.K., Curran W.J. (2017). Comparison of Concurrent Use of Thoracic Radiation with Either Carboplatin-Paclitaxel or Cisplatin-Etoposide for Patients with Stage III Non-Small-Cell Lung Cancer: A Systematic Review. JAMA Oncol..

[47] Zheng Q., Min S., Zhou Y. (2022). A network meta-analysis for efficacies and toxicities of different concurrent chemoradiotherapy regimens in the treatment of locally advanced non-small cell lung cancer. BMC Cancer.

[48] Kasmann L., Dietrich A., Staab-Weijnitz C.A., Manapov F., Behr J., Rimner A., Jeremic B., Senan S., De Ruysscher D., Lauber K. (2020). Radiation-induced lung toxicity–cellular and molecular mechanisms of pathogenesis, management, and literature review. Radiat. Oncol..

[49] Wang Y., Zhang T., Huang Y., Li W., Zhao J., Yang Y., Li C., Wang L., Bi N. (2022). Real-World Safety and Efficacy of Consolidation Durvalumab After Chemoradiation Therapy for Stage III Non-small Cell Lung Cancer: A Systematic Review and Meta-analysis. Int. J. Radiat. Oncol. Biol. Phys..

[50] Han C., Qiu J., Bai L., Liu T., Chen J., Wang H., Dang J. (2024). Pneumonitis Risk After Chemoradiotherapy with and without Immunotherapy in Patients with Locally Advanced Non-Small Cell Lung Cancer: A Systematic Review and Meta-Analysis. Int. J. Radiat. Oncol. Biol. Phys..

[51] Acker F., Reck M., Martin D., Rieken S., Heinzen S., Rost M., Aguinarte L., Schulte H., Serve H., Oellerich T. (2025). Efficacy and safety of immune checkpoint inhibition combined with concurrent chemoradiotherapy in patients with stage III unresectable non-small cell lung cancer: A systematic review and meta-analysis. Eur. J. Cancer.

[52] Chun S.G., Hu C., Komaki R.U., Timmerman R.D., Schild S.E., Bogart J.A., Dobelbower M.C., Bosch W., Kavadi V.S., Narayan S. (2024). Long-Term Prospective Outcomes of Intensity Modulated Radiotherapy for Locally Advanced Lung Cancer: A Secondary Analysis of a Randomized Clinical Trial. JAMA Oncol..

[53] Hou Z., Dong B., Yao Q., Chen H., Shao Q., Li M., Wang J., Chen K., Zhu Z., Peng F. (2025). Pirfenidone for grade 2 and grade 3 radiation-induced lung injury: A multicentre, open-label, randomised, phase 2 trial. Lancet Oncol..

[54] Herbach E., O’Rorke M.A., Carnahan R.M., McDowell B.D., Allen B., Grumbach I., London B., Smith B.J., Spitz D.R., Seaman A. (2022). Cardiac Adverse Events Associated with Chemo-Radiation Versus Chemotherapy for Resectable Stage III Non-Small-Cell Lung Cancer: A Surveillance, Epidemiology and End Results-Medicare Study. J. Am. Heart Assoc..

[55] Belzile-Dugas E., Eisenberg M.J. (2021). Radiation-Induced Cardiovascular Disease: Review of an Underrecognized Pathology. J. Am. Heart Assoc..

[56] Wallace N.D., Alexander M., Xie J., Ball D., Hegi-Johnson F., Plumridge N., Siva S., Shaw M., Harden S., John T. (2024). The impact of pre-treatment smoking status on survival after chemoradiotherapy for locally advanced non-small-cell lung cancer. Lung Cancer.

[57] Lyon A.R., Lopez-Fernandez T., Couch L.S., Asteggiano R., Aznar M.C., Bergler-Klein J., Boriani G., Cardinale D., Cordoba R., Cosyns B. (2022). 2022 ESC Guidelines on cardio-oncology developed in collaboration with the European Hematology Association (EHA), the European Society for Therapeutic Radiology and Oncology (ESTRO) and the International Cardio-Oncology Society (IC-OS). Eur. Heart J..

[58] Hendriks L.E., Kerr K.M., Menis J., Mok T.S., Nestle U., Passaro A., Peters S., Planchard D., Smit E.F., Solomon B.J. (2023). Oncogene-addicted metastatic non-small-cell lung cancer: ESMO Clinical Practice Guideline for diagnosis, treatment and follow-up. Ann. Oncol..

[59] Xu Y., Tong X., Yan J., Wu X., Shao Y.W., Fan Y. (2018). Short-Term Responders of Non-Small Cell Lung Cancer Patients to EGFR Tyrosine Kinase Inhibitors Display High Prevalence of TP53 Mutations and Primary Resistance Mechanisms. Transl. Oncol..

[60] Shalata W., Zolnoorian J., Migliozzi G., Jama A.A., Dudnik Y., Cohen A.Y., Meirovitz A., Yakobson A. (2023). Long-Lasting Therapeutic Response following Treatment with Pembrolizumab in Patients with Non-Small Cell Lung Cancer: A Real-World Experience. Int. J. Mol. Sci..

[61] Janne P.A., Planchard D., Kobayashi K., Yang J.C., Liu Y., Valdiviezo N., Kim T.M., Jiang L., Kagamu H., Yanagitani N. (2026). Survival with Osimertinib plus Chemotherapy in EGFR-Mutated Advanced NSCLC. N. Engl. J. Med..

[62] Yang J.C., Lu S., Hayashi H., Felip E., Spira A.I., Girard N., Kim Y.J., Lee S.H., Ostapenko Y., Danchaivijitr P. (2025). Overall Survival with Amivantamab-Lazertinib in EGFR-Mutated Advanced NSCLC. N. Engl. J. Med..

[63] Cho B.C., Li W., Spira A.I., Sauder M., Feldman J., Bozorgmehr F., Mak M., Smith J., Voon P.J., Liu B. (2025). Enhanced Versus Standard Dermatologic Management with Amivantamab-Lazertinib in EGFR-Mutated Advanced NSCLC: The COCOON Global Randomized Controlled Trial. J. Thorac. Oncol..

[64] Leighl N.B., Akamatsu H., Lim S.M., Cheng Y., Minchom A.R., Marmarelis M.E., Sanborn R.E., Chih-Hsin Yang J., Liu B., John T. (2024). Subcutaneous Versus Intravenous Amivantamab, Both in Combination with Lazertinib, in Refractory Epidermal Growth Factor Receptor-Mutated Non-Small Cell Lung Cancer: Primary Results From the Phase III PALOMA-3 Study. J. Clin. Oncol..

[65] Skoulidis F., Arbour K.C., Popat S., Lindsay C.R., Hironouchi H., Zalcman G., Curioni-Fontecedro A., Naidoo J., de Langen A.J., Mitra P. (2025). P3.18.65 Trial in Progress—RAsolve 301: A Phase 3 Study of Daraxonrasib (RMC-6236) Vs. Docetaxel in in Patients with Previously Treated RAS Mutant NSCLC. J. Thorac. Oncol..

[66] Punekar S.R., Hong D.S., Luo J., Ou S.H.I., Johnson M.L., Spira A., Garrido-Laguna I., Goldman J.W., Herzberg B., Saltos A. (2025). 6MO: Safety and clinical activity of daraxonrasib (RMC-6236) in RAS mutant non-small cell lung cancer (NSCLC). J. Thorac. Oncol..

[67] Solomon B.J., Liu G., Felip E., Mok T.S.K., Soo R.A., Mazieres J., Shaw A.T., de Marinis F., Goto Y., Wu Y.L. (2024). Lorlatinib Versus Crizotinib in Patients with Advanced ALK-Positive Non-Small Cell Lung Cancer: 5-Year Outcomes From the Phase III CROWN Study. J. Clin. Oncol..

[68] Cadranel J., Corre R., Roget P., Wingler M., Alliaume S., Bausson P., Capdevielle L., Mendes J., Mogenet A., Mennecier B. (2026). Living with ALK fusion-positive NSCLC: A French nationwide survey of the psychosocial impact among patients, caregivers and healthcare professionals. JTO Clin. Res. Rep..

[69] Barsouk A., Heidlauf A., Goel K., Rushkin L., Anran Huang A., Elghawy O., Yu C., Wang L., Yang D., Kurian M. (2026). Osimertinib dose-reduction and survival in 1L EGFR-mutated metastatic non-small cell lung cancer (mNSCLC). Lung Cancer.

[70] Liu G., Mazieres J., Stratmann J., Ou S.I., Mok T., Grizzard M., Goto Y., Felip E., Solomon B.J., Bauer T.M. (2024). A pragmatic guide for management of adverse events associated with lorlatinib. Lung Cancer.

[71] Hendriks L.E., Kerr K.M., Menis J., Mok T.S., Nestle U., Passaro A., Peters S., Planchard D., Smit E.F., Solomon B.J. (2023). Non-oncogene-addicted metastatic non-small-cell lung cancer: ESMO Clinical Practice Guideline for diagnosis, treatment and follow-up. Ann. Oncol..

[72] Reck M., Remon J., Hellmann M.D. (2022). First-Line Immunotherapy for Non-Small-Cell Lung Cancer. J. Clin. Oncol..

[73] Guerra C., Pressman A., Hurley P., Garrett-Mayer E., Bruinooge S.S., Howson A., Kaltenbaugh M., Hanley Williams J., Boehmer L., Bernick L.A. (2023). Increasing Racial and Ethnic Equity, Diversity, and Inclusion in Cancer Treatment Trials: Evaluation of an ASCO-Association of Community Cancer Centers Site Self-Assessment. JCO Oncol. Pract..

[74] Ricciuti B., Hong L., Gariazzo E., Gorria T., Thummalapalli R., Awosika N., Mollica L., Citarella F., Gandhi M., Elkrief A. (2024). P4.11E.17 Clinical Outcomes and Immunotherapy Retreatment in Patients with Metastatic NSCLC Who Complete at Least Two Years of Immune Checkpoint Blockade. J. Thorac. Oncol..

[75] Sun L., Bleiberg B., Hwang W.T., Marmarelis M.E., Langer C.J., Singh A., Cohen R.B., Mamtani R., Aggarwal C. (2023). Association Between Duration of Immunotherapy and Overall Survival in Advanced Non-Small Cell Lung Cancer. JAMA Oncol..

[76] Rousseau A., Michiels S., Simon-Tillaux N., Lolivier A., Bonastre J., Planchard D., Barlesi F., Remon J., Lavaud P., Aldea M. (2024). Impact of pembrolizumab treatment duration on overall survival and prognostic factors in advanced non-small cell lung cancer: A nationwide retrospective cohort study. Lancet Reg. Health–Eur..

[77] Gemelli M., Sala L., Morabito A., Sforza V., Cortellini A., La Cava G., Bertolini F., Guaitoli G., Carnio S., Novello S. (2026). Real-world outcomes of immunotherapy discontinuation at two years in advanced non-small cell lung cancer: Final results of the I-STOP study. Eur. J. Cancer.

[78] Elghawy O., Barsouk A., Sussman J.H., Bleiberg B.A., Reed-Guy L., D’Avella C., Singh A., Ciunci C., Robinson K., Kosteva J. (2025). Late Immune-Related Adverse Events After At Least Two Years of Immune Checkpoint Inhibitor Therapy: Incidence and Association with Survival in Patients with Advanced NSCLC. JTO Clin. Res. Rep..

[79] Xiong A., Wang L., Chen J., Wu L., Liu B., Yao J., Zhong H., Li J., Cheng Y., Sun Y. (2025). Ivonescimab versus pembrolizumab for PD-L1-positive non-small cell lung cancer (HARMONi-2): A randomised, double-blind, phase 3 study in China. Lancet.

[80] Investigators H.A.-A.S., Fang W., Zhao Y., Luo Y., Yang R., Huang Y., He Z., Zhao H., Li M., Li K. (2024). Ivonescimab Plus Chemotherapy in Non-Small Cell Lung Cancer with EGFR Variant: A Randomized Clinical Trial. JAMA.

[81] Mountzios G., Sun L., Cho B.C., Demirci U., Baka S., Gumus M., Lugini A., Zhu B., Yu Y., Korantzis I. (2025). Tarlatamab in Small-Cell Lung Cancer after Platinum-Based Chemotherapy. N. Engl. J. Med..

[82] Besse B., Felip E., Garcia Campelo R., Cobo M., Mascaux C., Madroszyk A., Cappuzzo F., Hilgers W., Romano G., Denis F. (2023). Randomized open-label controlled study of cancer vaccine OSE2101 versus chemotherapy in HLA-A2-positive patients with advanced non-small-cell lung cancer with resistance to immunotherapy: ATALANTE-1. Ann. Oncol..

[83] Horn L., Mansfield A.S., Szczesna A., Havel L., Krzakowski M., Hochmair M.J., Huemer F., Losonczy G., Johnson M.L., Nishio M. (2018). First-Line Atezolizumab plus Chemotherapy in Extensive-Stage Small-Cell Lung Cancer. N. Engl. J. Med..

[84] Paz-Ares L., Dvorkin M., Chen Y., Reinmuth N., Hotta K., Trukhin D., Statsenko G., Hochmair M.J., Ozguroglu M., Ji J.H. (2019). Durvalumab plus platinum-etoposide versus platinum-etoposide in first-line treatment of extensive-stage small-cell lung cancer (CASPIAN): A randomised, controlled, open-label, phase 3 trial. Lancet.

[85] Paz-Ares L., Borghaei H., Liu S.V., Peters S., Herbst R.S., Stencel K., Majem M., Sendur M.A.N., Czyzewicz G., Caro R.B. (2025). Efficacy and safety of first-line maintenance therapy with lurbinectedin plus atezolizumab in extensive-stage small-cell lung cancer (IMforte): A randomised, multicentre, open-label, phase 3 trial. Lancet.

[86] Dingemans A.C., Fruh M., Ardizzoni A., Besse B., Faivre-Finn C., Hendriks L.E., Lantuejoul S., Peters S., Reguart N., Rudin C.M. (2021). Small-cell lung cancer: ESMO Clinical Practice Guidelines for diagnosis, treatment and follow-up(☆). Ann. Oncol..

[87] Cheng Y., Spigel D.R., Cho B.C., Laktionov K.K., Fang J., Chen Y., Zenke Y., Lee K.H., Wang Q., Navarro A. (2024). Durvalumab after Chemoradiotherapy in Limited-Stage Small-Cell Lung Cancer. N. Engl. J. Med..

[88] Gondi V., Pugh S.L., Mehta M.P., Wefel J.S., Tome W.A., Sun A.Y., Grecula J., Redmond K.J., Fogh S., Gaspar L. (2025). Hippocampal Avoidance During Prophylactic Cranial Irradiation for Patients with Small Cell Lung Cancer: Randomized Phase II/III Trial NRG-CC003. J. Clin. Oncol..

[89] Caini S., Del Riccio M., Vettori V., Scotti V., Martinoli C., Raimondi S., Cammarata G., Palli D., Banini M., Masala G. (2022). Quitting Smoking At or Around Diagnosis Improves the Overall Survival of Lung Cancer Patients: A Systematic Review and Meta-Analysis. J. Thorac. Oncol..

[90] (2024). WHO Clinical Treatment Guideline for Tobacco Cessation in Adults [Internet].

[91] Burris J.L., Studts J.L., DeRosa A.P., Ostroff J.S. (2015). Systematic Review of Tobacco Use after Lung or Head/Neck Cancer Diagnosis: Results and Recommendations for Future Research. Cancer Epidemiol. Biomark. Prev..

[92] Cinciripini P.M., Kypriotakis G., Blalock J.A., Karam-Hage M., Beneventi D.M., Robinson J.D., Minnix J.A., Warren G.W. (2024). Survival Outcomes of an Early Intervention Smoking Cessation Treatment After a Cancer Diagnosis. JAMA Oncol..

[93] Ostroff J.S., Banerjee S.C., Lynch K., Shen M.J., Williamson T.J., Haque N., Riley K., Hamann H.A., Rigney M., Park B. (2022). Reducing stigma triggered by assessing smoking status among patients diagnosed with lung cancer: De-stigmatizing do and don’t lessons learned from qualitative interviews. PEC Innov..

[94] Lindson N., Livingstone-Banks J., Butler A.R., McRobbie H., Bullen C.R., Hajek P., Wu A.D., Begh R., Theodoulou A., Notley C. (2025). Electronic cigarettes for smoking cessation. Cochrane Database Syst. Rev..

[95] Tattan-Birch H., Hartmann-Boyce J., Kock L., Simonavicius E., Brose L., Jackson S., Shahab L., Brown J. (2022). Heated tobacco products for smoking cessation and reducing smoking prevalence. Cochrane Database Syst. Rev..

[96] Smith T.J., Temin S., Alesi E.R., Abernethy A.P., Balboni T.A., Basch E.M., Ferrell B.R., Loscalzo M., Meier D.E., Paice J.A. (2012). American Society of Clinical Oncology provisional clinical opinion: The integration of palliative care into standard oncology care. J. Clin. Oncol..

[97] Temel J.S., Petrillo L.A., Greer J.A. (2022). Patient-Centered Palliative Care for Patients with Advanced Lung Cancer. J. Clin. Oncol..

[98] Jordan R.I., Allsop M.J., ElMokhallalati Y., Jackson C.E., Edwards H.L., Chapman E.J., Deliens L., Bennett M.I. (2020). Duration of palliative care before death in international routine practice: A systematic review and meta-analysis. BMC Med..

[99] Temel J.S., Jackson V.A., El-Jawahri A., Rinaldi S.P., Petrillo L.A., Kumar P., McGrath K.A., LeBlanc T.W., Kamal A.H., Jones C.A. (2024). Stepped Palliative Care for Patients with Advanced Lung Cancer: A Randomized Clinical Trial. JAMA.

[100] Richard H.A., Sarathy R., Rabideau D.J., Feldman J., Cartagena L., Patel H., Sequist L.V., Park E., Jackson V., Greer J.A. (2025). Blended palliative and survivorship care intervention (POISE) for patients with metastatic oncogene-driven non-small cell lung cancer: Study protocol for a pilot randomised controlled trial. BMJ Open.

[101] Allende S., Turcott J.G., Verastegui E., Rodriguez-Mayoral O., Flores-Estrada D., Perez Camargo D.A., Ramos-Ramirez M., Martinez-Hernandez J.N., Onate-Ocana L.F., Pina P.S. (2024). Early Incorporation to Palliative Care (EPC) in Patients with Advanced Non-Small Cell Lung Cancer: The PACO Randomized Clinical Trial. Oncologist.

[102] O’Neill H., Robertson M., Kain D., Syed I., Pauli G., Parker C.M., Digby G.C. (2023). Improving Access and Timeliness of Early Palliative Care Specialist Assessment for Patients with Advanced Lung Cancer in a Rapid Assessment Clinic. J. Palliat. Med..

[103] (1998). Cancer care during the last phase of life. J. Clin. Oncol..

[104] Hooker E.R., Chapa J., Vranas K.C., Niederhausen M., Goodlin S.J., Slatore C.G., Sullivan D.R. (2023). Intersection of Palliative Care and Hospice Use Among Patients with Advanced Lung Cancer. J. Palliat. Med..

[105] Pihlaja H., Piili R.P., Nuutinen M., Saarto T., Lehto J.T., Carpen T. (2026). Specialist Palliative Care Reduces the Need for Acute Health Care Services in Patients with Nonmalignant Pulmonary Diseases and Lung Cancer: A Nationwide Study. J. Palliat. Med..

[106] Mack J.W., Cronin A., Keating N.L., Taback N., Huskamp H.A., Malin J.L., Earle C.C., Weeks J.C. (2012). Associations between end-of-life discussion characteristics and care received near death: A prospective cohort study. J. Clin. Oncol..

[107] Mack J.W., Cronin A., Taback N., Huskamp H.A., Keating N.L., Malin J.L., Earle C.C., Weeks J.C. (2012). End-of-life care discussions among patients with advanced cancer: A cohort study. Ann. Intern. Med..

[108] Epstein A.S., Prigerson H.G., O’Reilly E.M., Maciejewski P.K. (2016). Discussions of Life Expectancy and Changes in Illness Understanding in Patients with Advanced Cancer. J. Clin. Oncol..

[109] Martin A., Carton M., Thery L., Burnod A., Daniel C., Du Rusquec P., Girard N., Bouleuc C. (2024). Palliative care integration and end-of-life care intensity for patients with NSCLC. Lung Cancer.

[110] Seow H., Barbera L.C., McGrail K., Burge F., Guthrie D.M., Lawson B., Chan K.K.W., Peacock S.J., Sutradhar R. (2022). Effect of Early Palliative Care on End-of-Life Health Care Costs: A Population-Based, Propensity Score-Matched Cohort Study. JCO Oncol. Pract..

[111] Parikh R.B., Ferrell W.J., Li Y., Chen J., Bilbrey L., Johnson N., White J., Sedhom R., Dickson N.R., Schleicher S. (2025). Algorithm-Based Palliative Care in Patients with Cancer: A Cluster Randomized Clinical Trial. JAMA Netw. Open.

[112] Bonsignore L., Bloom N., Steinhauser K., Nichols R., Allen T., Twaddle M., Bull J. (2018). Evaluating the Feasibility and Acceptability of a Telehealth Program in a Rural Palliative Care Population: TapCloud for Palliative Care. J. Pain Symptom Manag..

[113] Saeidzadeh S., Kamalumpundi V., Chi N.C., Nair R., Gilbertson-White S. (2021). Web and mobile-based symptom management interventions for physical symptoms of people with advanced cancer: A systematic review and meta-analysis. Palliat. Med..

[114] Boulanger M.C., Lo S.B., Centracchio J.A., Jewett B., Freese M., Holtze M., Jacobs J.M., Petrillo L.A., Bauman J., El-Jawahri A. (2025). Pilot Feasibility Trial of a Supportive Care Digital Application for Patients with Advanced Nonsmall Cell Lung Cancer. J. Palliat. Med..

[115] Islam J.Y., Braithwaite D., Zhang D., Guo Y., Tailor T.D., Akinyemiju T. (2023). Racial and ethnic inequities of palliative care use among advanced Non-Small cell lung cancer patients in the US. Cancer Med..

[116] Serna M.K., Polychronopoulou E., Rodriguez A., Ramachandran A., Goodrich M., O’Mahoney S., Raji M., Kuo Y.F. (2026). Sex and Racial/Ethnic Differences in End-of-Life Care in Decedents with Lung Cancer in Texas. J. Palliat. Med..

[117] Khullar K., Plascak J.J., Habib M.H., Nagengast S., Parikh R.R. (2024). Extensive stage small cell lung cancer (ES-SCLC) and palliative care disparities: A national cancer database study. BMJ Support. Palliat. Care.

[118] Moore S.C., Lee I.M., Weiderpass E., Campbell P.T., Sampson J.N., Kitahara C.M., Keadle S.K., Arem H., Berrington de Gonzalez A., Hartge P. (2016). Association of Leisure-Time Physical Activity with Risk of 26 Types of Cancer in 1.44 Million Adults. JAMA Intern. Med..

[119] Strasser B., Steindorf K., Wiskemann J., Ulrich C.M. (2013). Impact of resistance training in cancer survivors: A meta-analysis. Med. Sci. Sports Exerc..

[120] Gerritsen J.K., Vincent A.J. (2016). Exercise improves quality of life in patients with cancer: A systematic review and meta-analysis of randomised controlled trials. Br. J. Sports Med..

[121] Quist M., Langer S.W., Lillelund C., Winther L., Laursen J.H., Christensen K.B., Rorth M., Adamsen L. (2020). Effects of an exercise intervention for patients with advanced inoperable lung cancer undergoing chemotherapy: A randomized clinical trial. Lung Cancer.

[122] Jensen S., Bloch Z., Quist M., Hansen T.T.D., Johansen C., Pappot H., Suetta C., Skjodt Rafn B. (2023). Sarcopenia and loss of muscle mass in patients with lung cancer undergoing chemotherapy treatment: A systematic review and meta-analysis. Acta Oncol..

[123] Bloch Z., Jensen S., Sorensen V., Langer S.W., Quist M. (2025). The Effect of Exercise on Quality of Life in Patients with Advanced Lung Cancer: A Secondary Analysis of a Randomized Controlled Trial. Clin. Lung Cancer.

[124] Toohey K., Chapman M., Rushby A.M., Urban K., Ingham G., Singh B. (2023). The effects of physical exercise in the palliative care phase for people with advanced cancer: A systematic review with meta-analysis. J. Cancer Surviv..

[125] Kim Y.W., Kwak K.I., Choi A.R., Park E.J., Lee B.J., Lee Y.J., Lee C.T. (2025). Association of Pre- and Postdiagnosis Physical Activity, Promotion and Maintenance with Lung Cancer Survival: A Nationwide Cohort Study. J. Cachexia Sarcopenia Muscle.

[126] Friedenreich C.M., Cook L.S., Wang Q., Kokts-Porietis R.L., McNeil J., Ryder-Burbidge C., Courneya K.S. (2020). Prospective Cohort Study of Pre- and Postdiagnosis Physical Activity and Endometrial Cancer Survival. J. Clin. Oncol..

[127] Sloan J.A., Cheville A.L., Liu H., Novotny P.J., Wampfler J.A., Garces Y.I., Clark M.M., Yang P. (2016). Impact of self-reported physical activity and health promotion behaviors on lung cancer survivorship. Health Qual. Life Outcomes.

[128] Rock C.L., Thomson C.A., Sullivan K.R., Howe C.L., Kushi L.H., Caan B.J., Neuhouser M.L., Bandera E.V., Wang Y., Robien K. (2022). American Cancer Society nutrition and physical activity guideline for cancer survivors. CA Cancer J. Clin..

[129] Ligibel J.A., Bohlke K., May A.M., Clinton S.K., Demark-Wahnefried W., Gilchrist S.C., Irwin M.L., Late M., Mansfield S., Marshall T.F. (2022). Exercise, Diet, and Weight Management During Cancer Treatment: ASCO Guideline. J. Clin. Oncol..

[130] Patel A.V., Friedenreich C.M., Moore S.C., Hayes S.C., Silver J.K., Campbell K.L., Winters-Stone K., Gerber L.H., George S.M., Fulton J.E. (2019). American College of Sports Medicine Roundtable Report on Physical Activity, Sedentary Behavior, and Cancer Prevention and Control. Med. Sci. Sports Exerc..

[131] Kennedy M.A., Bayes S., Newton R.U., Zissiadis Y., Spry N.A., Taaffe D.R., Hart N.H., Galvao D.A. (2022). Implementation barriers to integrating exercise as medicine in oncology: An ecological scoping review. J. Cancer Surviv..

[132] Avancini A., Trestini I., Tregnago D., Belluomini L., Sposito M., Insolda J., Schena F., Milella M., Pilotto S. (2023). Willingness, preferences, barriers, and facilitators of a multimodal supportive care intervention including exercise, nutritional and psychological approach in patients with cancer: A cross-sectional study. J. Cancer Res. Clin. Oncol..

[133] Avancini A., Giannarelli D., Borsati A., Carnio S., Cantale O., Nepote A., Mangiapane F., Bafunno D., Galetta D., Longo V. (2024). A cross-sectional study evaluating the exercise discussion with oncologist during cancer consultation: The CONNECT study. ESMO Open.

[134] Muscaritoli M., Arends J., Bachmann P., Baracos V., Barthelemy N., Bertz H., Bozzetti F., Hutterer E., Isenring E., Kaasa S. (2021). ESPEN practical guideline: Clinical Nutrition in cancer. Clin. Nutr..

[135] Zhang F.F., Liu S., John E.M., Must A., Demark-Wahnefried W. (2015). Diet quality of cancer survivors and noncancer individuals: Results from a national survey. Cancer.

[136] Fearon K., Strasser F., Anker S.D., Bosaeus I., Bruera E., Fainsinger R.L., Jatoi A., Loprinzi C., MacDonald N., Mantovani G. (2011). Definition and classification of cancer cachexia: An international consensus. Lancet Oncol..

[137] Al-Sawaf O., Weiss J., Skrzypski M., Lam J.M., Karasaki T., Zambrana F., Kidd A.C., Frankell A.M., Watkins T.B.K., Martinez-Ruiz C. (2023). Body composition and lung cancer-associated cachexia in TRACERx. Nat. Med..

[138] Yue M., Qin Z., Hu L., Ji H. (2024). Understanding cachexia and its impact on lung cancer and beyond. Chin. Med. J. Pulm. Crit. Care Med..

[139] Schneider B.J., Naidoo J., Santomasso B.D., Lacchetti C., Adkins S., Anadkat M., Atkins M.B., Brassil K.J., Caterino J.M., Chau I. (2021). Management of Immune-Related Adverse Events in Patients Treated with Immune Checkpoint Inhibitor Therapy: ASCO Guideline Update. J. Clin. Oncol..

[140] Peters S., Gadgeel S.M., Mok T., Nadal E., Kilickap S., Swalduz A., Cadranel J., Sugawara S., Chiu C.H., Yu C.J. (2024). Entrectinib in ROS1-positive advanced non-small cell lung cancer: The phase 2/3 BFAST trial. Nat. Med..

[141] Avancini A., Sposito M., Adamoli G., Borsati A., Ciurnelli C., Scaglione I.M., Eccher S., Toniolo L., Tregnago D., Longo L. (2025). Impact of Long-Term Structured Exercise on Body Composition in an NTRK Fusion-Positive NSCLC Patient Treated with Entrectinib. Thorac. Cancer.

[142] Bauer T.M., Felip E., Solomon B.J., Thurm H., Peltz G., Chioda M.D., Shaw A.T. (2019). Clinical Management of Adverse Events Associated with Lorlatinib. Oncologist.

[143] Mantz L., Mercaldo N.D., Peterson J., Abadi P.T., Krueger E.A., Fintelmann F.J., Dagogo-Jack I. (2026). Weight Gain on Lorlatinib is Associated with Accumulation of Visceral Adipose Tissue. J. Thorac. Oncol..

[144] Sullivan E.S., Rice N., Kingston E., Kelly A., Reynolds J.V., Feighan J., Power D.G., Ryan A.M. (2021). A national survey of oncology survivors examining nutrition attitudes, problems and behaviours, and access to dietetic care throughout the cancer journey. Clin. Nutr. ESPEN.

[145] Keaver L., Richmond J., Rafferty F., Douglas P. (2023). Sources of nutrition advice and desired nutrition guidance in oncology care: Patient’s perspectives. J. Hum. Nutr. Diet..

[146] Scott E.C.S., Hoskin P.J. (2024). Health inequalities in cancer care: A literature review of pathways to diagnosis in the United Kingdom. EClinicalMedicine.

[147] Ryding H.G., Rigby R.R., Johnston E.A., Kruger R., Mitchell L.J. (2025). Dietitians’ practices and perspectives of the delivery of nutritional care to cancer survivors in the primary care setting. Support. Care Cancer.

[148] Rauma V., Sintonen H., Rasanen J.V., Salo J.A., Ilonen I.K. (2015). Long-term lung cancer survivors have permanently decreased quality of life after surgery. Clin. Lung Cancer.

[149] Cochrane A., Woods S., Dunne S., Gallagher P. (2022). Unmet supportive care needs associated with quality of life for people with lung cancer: A systematic review of the evidence 2007–2020. Eur. J. Cancer Care.

[150] Haun M.W., Sklenarova H., Villalobos M., Thomas M., Brechtel A., Lowe B., Herzog W., Hartmann M. (2014). Depression, anxiety and disease-related distress in couples affected by advanced lung cancer. Lung Cancer.

[151] Hirpara D.H., Gupta V., Davis L.E., Zhao H., Hallet J., Mahar A.L., Sutradhar R., Doherty M., Louie A.V., Kidane B. (2020). Severe symptoms persist for Up to one year after diagnosis of stage I-III lung cancer: An analysis of province-wide patient reported outcomes. Lung Cancer.

[152] Bauml J.M., Troxel A., Epperson C.N., Cohen R.B., Schmitz K., Stricker C., Shulman L.N., Bradbury A., Mao J.J., Langer C.J. (2016). Scan-associated distress in lung cancer: Quantifying the impact of “scanxiety”. Lung Cancer.

[153] Derry-Vick H.M., Heathcote L.C., Glesby N., Stribling J., Luebke M., Epstein A.S., Prigerson H.G. (2023). Scanxiety among Adults with Cancer: A Scoping Review to Guide Research and Interventions. Cancers.

[154] Hu Y., Xiao L.D., Tang C., Cao W., Wang Y. (2024). Prevalence and risk factors of sleep disturbances among patients with lung cancer: Systematic review and meta-analysis. Support. Care Cancer.

[155] Nissen E.R., Neumann H., Knutzen S.M., Henriksen E.N., Amidi A., Johansen C., von Heymann A., Christiansen P., Zachariae R. (2024). Interventions for insomnia in cancer patients and survivors-a comprehensive systematic review and meta-analysis. JNCI Cancer Spectr..

[156] Sanft T., Day A., Ansbaugh S., Armenian S., Baker K.S., Ballinger T., Demark-Wahnefried W., Dickinson K., Fairman N.P., Felciano J. (2023). NCCN Guidelines^®^ Insights: Survivorship, Version 1.2023: Featured updates to the NCCN guidelines. J. Natl. Compr. Cancer Netw..

[157] Andersen B.L., Lacchetti C., Ashing K., Berek J.S., Berman B.S., Bolte S., Dizon D.S., Given B., Nekhlyudov L., Pirl W. (2023). Management of Anxiety and Depression in Adult Survivors of Cancer: ASCO Guideline Update. J. Clin. Oncol..

[158] Zhong C., Luo X., Tan M., Chi J., Guo B., Tang J., Guo Z., Deng S., Zhang Y., Wu Y. (2025). Digital Health Interventions to Improve Mental Health in Patients with Cancer: Umbrella Review. J. Med. Internet Res..

[159] Loi M., Wortel R.C., Francolini G., Incrocci L. (2019). Sexual Function in Patients Treated with Stereotactic Radiotherapy For Prostate Cancer: A Systematic Review of the Current Evidence. J. Sex. Med..

[160] Tramacere F., Lancellotta V., Casa C., Fionda B., Cornacchione P., Mazzarella C., De Vincenzo R.P., Macchia G., Ferioli M., Rovirosa A. (2022). Assessment of Sexual Dysfunction in Cervical Cancer Patients after Different Treatment Modality: A Systematic Review. Medicina.

[161] McCabe M.P., Sharlip I.D., Lewis R., Atalla E., Balon R., Fisher A.D., Laumann E., Lee S.W., Segraves R.T. (2016). Incidence and Prevalence of Sexual Dysfunction in Women and Men: A Consensus Statement from the Fourth International Consultation on Sexual Medicine 2015. J. Sex. Med..

[162] Perz J., Ussher J.M., Gilbert E., Australian C., Sexuality Study T. (2014). Feeling well and talking about sex: Psycho-social predictors of sexual functioning after cancer. BMC Cancer.

[163] Ospina-Serrano A.V., Maximiano-Alonso C., Guillen-Sentis P., Losada B., Cruz-Castellanos P., Lopez-Castro R., Campos-Balea B., Azkona-Uribelarrea E., Cardena A., Valdivia A. (2026). Multidisciplinary Approach to Sexual Dysfunction in Persons with Lung Cancer-Spanish Lung Cancer Group Expert Consensus Statement. JTO Clin. Res. Rep..

[164] Bober S.L., Varela V.S. (2012). Sexuality in adult cancer survivors: Challenges and intervention. J. Clin. Oncol..

[165] Zanni L., Fasse L., Letot J., Fromage M.N., Flahault C. (2025). Sexual health of patients with lung cancer: A systematic review. Support. Care Cancer.

[166] Bolat M.S., Celik B., Celik H.K., Akdeniz E. (2019). The impact of thoracotomy on psychological and sexual function in men with lung cancer. Rev. Int. Androl..

[167] Costa M., Fernandes C.L., Leite J., Vilaca M., Estevinho F., Magalhaes H. (2025). Sexual Health of Patients in Treatment for Lung Cancer: An Undercover Concern for Patients and Oncologists. Curr. Oncol..

[168] Ospina-Serrano A.V., Collazo-Lorduy A., Azkona-Uribelarrea E., Guillen-Sentis P., Aparisi F., Lopez R., Cruz P., Valdivia A., Cordeiro P., Olivares-Hernandez A. (2025). LUDICAS: Sexual dysfunction in patients with lung cancer, a multicenter cross-sectional study. ESMO Open.

[169] Lindau S.T., Surawska H., Paice J., Baron S.R. (2011). Communication about sexuality and intimacy in couples affected by lung cancer and their clinical-care providers. Psychooncology.

[170] Florez N., Kiel L., Meza K., Wei Z., Mazzola E., Velazquez A.I., Franco I., Fidler M.J., Elkins I., Feldman J. (2024). Sexual Health Assessment in Women with Lung Cancer study: Sexual health assessment in women with lung cancer. Cancer.

[171] Ospina-Serrano A.V., Maximiano C., Cantos B., Torrente M., Mendez M., Sanchez J.C., Calvo V., Collazo-Lorduy A., Blanco M., Nunez B. (2024). Sexual dysfunction in patients with cancer, a challenge in oncology practice: Results of the CLARIFY project. Clin. Transl. Oncol..

[172] Krouwel E.M., Albers L.F., Nicolai M.P.J., Putter H., Osanto S., Pelger R.C.M., Elzevier H.W. (2020). Discussing Sexual Health in the Medical Oncologist’s Practice: Exploring Current Practice and Challenges. J. Cancer Educ..

[173] Carter J., Lacchetti C., Andersen B.L., Barton D.L., Bolte S., Damast S., Diefenbach M.A., DuHamel K., Florendo J., Ganz P.A. (2018). Interventions to Address Sexual Problems in People with Cancer: American Society of Clinical Oncology Clinical Practice Guideline Adaptation of Cancer Care Ontario Guideline. J. Clin. Oncol..

[174] Jackson S.E., Wardle J., Steptoe A., Fisher A. (2016). Sexuality after a cancer diagnosis: A population-based study. Cancer.

[175] Cramp F., Byron-Daniel J. (2012). Exercise for the management of cancer-related fatigue in adults. Cochrane Database Syst. Rev..

[176] Bower J.E., Lacchetti C., Alici Y., Barton D.L., Bruner D., Canin B.E., Escalante C.P., Ganz P.A., Garland S.N., Gupta S. (2024). Management of Fatigue in Adult Survivors of Cancer: ASCO-Society for Integrative Oncology Guideline Update. J. Clin. Oncol..

[177] Pang K., Pan D., Xu H., Ma Y., Wang J., Xu P., Wang H., Zang G. (2022). Advances in physical diagnosis and treatment of male erectile dysfunction. Front. Physiol..

[178] Carter J., Stabile C., Seidel B., Baser R.E., Goldfarb S., Goldfrank D.J. (2017). Vaginal and sexual health treatment strategies within a female sexual medicine program for cancer patients and survivors. J. Cancer Surviv..

[179] Su H.I., Lacchetti C., Letourneau J., Partridge A.H., Qamar R., Quinn G.P., Reinecke J., Smith J.F., Tesch M., Wallace W.H. (2025). Fertility Preservation in People with Cancer: ASCO Guideline Update. J. Clin. Oncol..

[180] Keller S.R., Rosen A., Lewis M.A., Park H.K., Babyak R., Feldman J., Ye F., Agarwal R., Ciombor K.K., Geiger T.M. (2024). Patient-Reported Discussions on Fertility Preservation Before Early-Onset Cancer Treatment. JAMA Netw. Open.

[181] Garutti M., Lambertini M., Puglisi F. (2021). Checkpoint inhibitors, fertility, pregnancy, and sexual life: A systematic review. ESMO Open.

[182] Duma N., Lambertini M. (2020). It Is Time to Talk about Fertility and Immunotherapy. Oncologist.

[183] Rambhatla A., Strug M.R., De Paredes J.G., Cordoba Munoz M.I., Thakur M. (2021). Fertility considerations in targeted biologic therapy with tyrosine kinase inhibitors: A review. J. Assist. Reprod. Genet..

[184] da Rosa P.R.A., De Cesaro M.P., Pereira Dau A.M., Duggavathi R., Bordignon V., Goncalves P.B.D. (2017). Reversible meiotic arrest of bovine oocytes by EGFR inhibition and follicular hemisections. Theriogenology.

[185] Duffin K., Mitchell R.T., Brougham M.F.H., Hamer G., van Pelt A.M.M., Mulder C.L. (2024). Impacts of cancer therapy on male fertility: Past and present. Mol. Asp. Med..

[186] Ozdemir B.C. (2021). Immune checkpoint inhibitor-related hypogonadism and infertility: A neglected issue in immuno-oncology. J. Immunother. Cancer.

[187] Laguna J.C., Tagliamento M., Lambertini M., Hiznay J., Mezquita L. (2024). Tackling Non-Small Cell Lung Cancer in Young Adults: From Risk Factors and Genetic Susceptibility to Lung Cancer Profile and Outcomes. Am. Soc. Clin. Oncol. Educ. Book.

[188] Florez N., Horiguchi M., Abioye O., Kaufman R., Haradon D., Walton S., Lederman R., Morabito A., Hebert A., Camidge R. (2024). P3.03I.19 The International Pregnancy and Lung Cancer Registry at Dana-Farber Cancer Institute. J. Thorac. Oncol..

[189] Allemani C., Matsuda T., Di Carlo V., Harewood R., Matz M., Niksic M., Bonaventure A., Valkov M., Johnson C.J., Esteve J. (2018). Global surveillance of trends in cancer survival 2000-14 (CONCORD-3): Analysis of individual records for 37,513,025 patients diagnosed with one of 18 cancers from 322 population-based registries in 71 countries. Lancet.

[190] Pallis A.G., Gridelli C., Wedding U., Faivre-Finn C., Veronesi G., Jaklitsch M., Luciani A., O’Brien M. (2014). Management of elderly patients with NSCLC; updated expert’s opinion paper: EORTC Elderly Task Force, Lung Cancer Group and International Society for Geriatric Oncology. Ann. Oncol..

[191] Bergerot C.D., Temin S., Verduzco-Aguirre H.C., Aapro M.S., Alibhai S.M.H., Aziz Z., de Celis Herrero M.C.P., Basu T., Extermann M., Kanesvaran R. (2025). Geriatric Assessment: ASCO Global Guideline. JCO Glob. Oncol..

[192] Kim D.H., Rockwood K. (2024). Frailty in Older Adults. N. Engl. J. Med..

[193] Dale W., Klepin H.D., Williams G.R., Alibhai S.M.H., Bergerot C., Brintzenhofeszoc K., Hopkins J.O., Jhawer M.P., Katheria V., Loh K.P. (2023). Practical Assessment and Management of Vulnerabilities in Older Patients Receiving Systemic Cancer Therapy: ASCO Guideline Update. J. Clin. Oncol..

[194] Wang S.C., Peng L. (2025). Frailty as a predictor of mortality in lung cancer survivors: Evidence from a nationally representative cohort NHIS 1997–2018. Sci. Rep..

[195] Grogan M., Hoyd R., Benedict J., Janse S., Williams N., Naughton M., Burd C.E., Paskett E.D., Rosko A., Spakowicz D.J. (2024). The FITNESS study: Longitudinal geriatric assessment, treatment toxicity, and biospecimen collection to assess functional disability among older adults with lung cancer. Front. Aging.

[196] Rached L., Geraud A., Frelaut M., Ap Thomas Z., Goldschmidt V., Beraud-Chaulet G., Nagera-Lazarovici C., Danlos F.X., Henon C., Parisi C. (2024). Antibody drug conjugates in older patients: State of the art. Crit. Rev. Oncol. Hematol..

[197] Tagliamento M., Frelaut M., Baldini C., Naigeon M., Nencioni A., Chaput N., Besse B. (2022). The use of immunotherapy in older patients with advanced non-small cell lung cancer. Cancer Treat. Rev..

[198] Taha A., Vinograd I., Sakhnini A., Eliakim-Raz N., Farbman L., Baslo R., Stemmer S.M., Gafter-Gvili A., Leibovici L., Paul M. (2015). The association between infections and chemotherapy interruptions among cancer patients: Prospective cohort study. J. Infect..

[199] Schmedt N., Heuer O.D., Hackl D., Sato R., Theilacker C. (2019). Burden of community-acquired pneumonia, predisposing factors and health-care related costs in patients with cancer. BMC Health Serv. Res..

[200] Kamboj M., Bohlke K., Baptiste D.M., Dunleavy K., Fueger A., Jones L., Kelkar A.H., Law L.Y., LeFebvre K.B., Ljungman P. (2024). Vaccination of Adults with Cancer: ASCO Guideline. J. Clin. Oncol..

[201] Leduc C., Antoni D., Charloux A., Falcoz P.E., Quoix E. (2017). Comorbidities in the management of patients with lung cancer. Eur. Respir. J..

[202] Gordon L.G., Merollini K.M.D., Lowe A., Chan R.J. (2017). A Systematic Review of Financial Toxicity Among Cancer Survivors: We Can’t Pay the Co-Pay. Patient.

[203] Yabroff K.R., Doran J.F., Zhao J., Chino F., Shih Y.T., Han X., Zheng Z., Bradley C.J., Bryant M.F. (2024). Cancer diagnosis and treatment in working-age adults: Implications for employment, health insurance coverage, and financial hardship in the United States. CA Cancer J. Clin..

[204] McLouth L.E., Nightingale C.L., Levine B.J., Burris J.L., McDougall J.A., Lycan T.W., Gabbard J., Ruiz J., Farris M., Blackstock A.W. (2021). Unmet Care Needs and Financial Hardship in Patients With Metastatic Non-Small-Cell Lung Cancer on Immunotherapy or Chemoimmunotherapy in Clinical Practice. JCO Oncol. Pract..

[205] Hsu M.L., Boulanger M.C., Olson S., Eaton C., Prichett L., Guo M., Miller M., Brahmer J., Forde P.M., Marrone K.A. (2024). Unmet Needs, Quality of Life, and Financial Toxicity Among Survivors of Lung Cancer. JAMA Netw. Open.

[206] Smith G.L., Banegas M.P., Acquati C., Chang S., Chino F., Conti R.M., Greenup R.A., Kroll J.L., Liang M.I., Pisu M. (2022). Navigating financial toxicity in patients with cancer: A multidisciplinary management approach. CA Cancer J. Clin..

[207] Jia S., Cui Y., Cheung D.S.T., Chau P.H., Lin C.C. (2026). Financial Hardship Among Newly Diagnosed Patients with Lung Cancer and Their Caregivers: A Dyadic Analysis. Semin. Oncol. Nurs..

[208] Fujinami R., Sun V., Zachariah F., Uman G., Grant M., Ferrell B. (2015). Family caregivers’ distress levels related to quality of life, burden, and preparedness. Psychooncology.

[209] Milbury K., Badr H., Fossella F., Pisters K.M., Carmack C.L. (2013). Longitudinal associations between caregiver burden and patient and spouse distress in couples coping with lung cancer. Support. Care Cancer.

[210] Ullrich A., Ascherfeld L., Marx G., Bokemeyer C., Bergelt C., Oechsle K. (2017). Quality of life, psychological burden, needs, and satisfaction during specialized inpatient palliative care in family caregivers of advanced cancer patients. BMC Palliat. Care.

[211] Hirayama K., Kuribara T., Oshikiri M. (2023). Experiences of the older spousal caregivers of patients with cancer during palliative chemotherapy: A qualitative descriptive study. BMC Palliat. Care.

[212] Murray S.A., Kendall M., Boyd K., Grant L., Highet G., Sheikh A. (2010). Archetypal trajectories of social, psychological, and spiritual wellbeing and distress in family care givers of patients with lung cancer: Secondary analysis of serial qualitative interviews. BMJ.

[213] Jassem J., Penrod J.R., Goren A., Gilloteau I. (2015). Caring for relatives with lung cancer in Europe: An evaluation of caregivers’ experience. Qual. Life Res..

[214] Grosse J., Treml J., Kersting A. (2018). Impact of caregiver burden on mental health in bereaved caregivers of cancer patients: A systematic review. Psychooncology.

[215] Tay D.L., Dubose K., Chipman J., Ellington L., Alnajar M., Iacob E., Stephens C., Ornstein K.A. (2025). Risk of mental health conditions in bereavement: A population-based analysis of lung cancer spouses. Front. Public Health.

[216] Lei F., Lee E., Shin J., Lee S.Y. (2023). Non-pharmacological interventions on anxiety and depression in lung cancer patients’ informal caregivers: A systematic review and meta-analysis. PLoS ONE.

[217] Cochrane A., Reid O., Woods S., Gallagher P., Dunne S. (2021). Variables associated with distress amongst informal caregivers of people with lung cancer: A systematic review of the literature. Psychooncology.

[218] Ihrig A., Karschuck P., Haun M.W., Thomas C., Huber J. (2020). Online peer-to-peer support for persons affected by prostate cancer: A systematic review. Patient Educ. Couns..

[219] Hamann H.A., Williamson T.J., Studts J.L., Ostroff J.S. (2021). Lung Cancer Stigma Then and Now: Continued Challenges Amid a Landscape of Progress. J. Thorac. Oncol..

[220] Price S.N., Shen M., Rigney M., Ostroff J.S., Hamann H.A. (2022). Identifying Barriers to Advocacy Among Patients with Lung Cancer: The Role of Stigma-Related Interpersonal Constraint. Oncol. Nurs. Forum.

[221] Passiglia F., Listi A., Bironzo P., Merlini A., Benso F., Napoli F., Barbu F.A., Zambelli V., Tabbo F., Reale M.L. (2025). Actionable NSCLC Mutation Identification by Comprehensive Genomic Profiling for Clinical Trial Enrollment: The European Program for the Routine Testing of Patients with Advanced Lung Cancer (EPROPA). J. Thorac. Oncol..

[222] Pompili C., Ungaro A., Pateli-Bell K., Shilo S., Hennink M., Diaz Y., Vallone S., Novello S., Montague D. (2026). Patient Advocacy in Transforming Surgical Lung Cancer Care in Europe. Interdiscip. Cardiovasc. Thorac. Surg..

[223] Dy S.M., Janssen E.M., Ferris A., Bridges J.F. (2017). Live, Learn, Pass It on: A Patient Advocacy Engagement Project on the Lived Experience of Lung Cancer Survivors. J. Patient Exp..

[224] Studts J.L., Carter-Bawa L., Hamann H.A., Smith R.A., Kazerooni E.A., Rosenthal L.S., Stigma and Nihilism Task Group of the American Cancer Society National Lung Cancer Roundtable (2025). The American Cancer Society National Lung Cancer Roundtable strategic plan: Changing the lung cancer story: Addressing survivorship, stigma, and nihilism to facilitate transformation. Cancer.

[225] Kurzrock R., Chaudhuri A.A., Feller-Kopman D., Florez N., Gorden J., Wistuba I.I. (2024). Healthcare disparities, screening, and molecular testing in the changing landscape of non-small cell lung cancer in the United States: A review. Cancer Metastasis Rev..

[226] Wagle N.S., Nogueira L., Devasia T.P., Mariotto A.B., Yabroff K.R., Islami F., Jemal A., Alteri R., Ganz P.A., Siegel R.L. (2025). Cancer treatment and survivorship statistics, 2025. CA Cancer J. Clin..

[227] Lai-Kwon J., Heynemann S., Flore J., Dhillon H., Duffy M., Burke J., Briggs L., Leigh L., Mileshkin L., Solomon B. (2021). Living with and beyond metastatic non-small cell lung cancer: The survivorship experience for people treated with immunotherapy or targeted therapy. J. Cancer Surviv..

[228] Lai-Kwon J., Heynemann S., Hart N.H., Chan R.J., Smith T.J., Nekhlyudov L., Jefford M. (2023). Evolving Landscape of Metastatic Cancer Survivorship: Reconsidering Clinical Care, Policy, and Research Priorities for the Modern Era. J. Clin. Oncol..

[229] Sheikh M., Mukeriya A., Shangina O., Brennan P., Zaridze D. (2021). Postdiagnosis Smoking Cessation and Reduced Risk for Lung Cancer Progression and Mortality: A Prospective Cohort Study. Ann. Intern. Med..

[230] Choi E., Luo S.J., Aredo J.V., Backhus L.M., Wilkens L.R., Su C.C., Neal J.W., Le Marchand L., Cheng I., Wakelee H.A. (2022). The Survival Impact of Second Primary Lung Cancer in Patients with Lung Cancer. J. Natl. Cancer Inst..

[231] Rabe B.J., Stafford J.W., Hassinger A.D., Swartzwelder H.S., Shofer S.L. (2022). Implementation and Effectiveness of a Veterans Affairs-Based Comprehensive Lung Cancer Survivorship Program. J. Cardiopulm. Rehabil. Prev..

[232] Price S.N., Willis A.R., Hensley A., Hyson J., Sohl S.J., D’Agostino R.B., Farris M., Petty W.J., de Hoyos A., Weaver K.E. (2025). Implementation and Retrospective Examination of a Lung Cancer Survivorship Clinic in a Comprehensive Cancer Center. Clin. Lung Cancer.

[233] Granger C.L., Edbrooke L., Antippa P., Wright G., McDonald C.F., Zannino D., Abo S., Krishnasamy M., Irving L., Lamb K.E. (2024). Home-Based Exercise and Self-Management After Lung Cancer Resection: A Randomized Clinical Trial. JAMA Netw. Open.

[234] Ha D.M., Comer A., Dollar B., Bedoy R., Ford M., Gozansky W.S., Zeng C., Arch J.J., Leach H.J., Malhotra A. (2023). Telemedicine-based inspiratory muscle training and walking promotion with lung cancer survivors following curative intent therapy: A parallel-group pilot randomized trial. Support. Care Cancer.

[235] van den Hurk C.J.G., Mols F., Eicher M., Chan R.J., Becker A., Geleijnse G., Walraven I., Coolbrandt A., Lustberg M., Velikova G. (2022). A Narrative Review on the Collection and Use of Electronic Patient-Reported Outcomes in Cancer Survivorship Care with Emphasis on Symptom Monitoring. Curr. Oncol..

[236] Basch E., Rocque G., Mody G., Mullangi S., Patt D. (2023). Tenets for Implementing Electronic Patient-Reported Outcomes for Remote Symptom Monitoring During Cancer Treatment. JCO Clin. Cancer Inform..

[237] Chaudhuri A.A., Chabon J.J., Lovejoy A.F., Newman A.M., Stehr H., Azad T.D., Khodadoust M.S., Esfahani M.S., Liu C.L., Zhou L. (2017). Early Detection of Molecular Residual Disease in Localized Lung Cancer by Circulating Tumor DNA Profiling. Cancer Discov..

[238] Pascual J., Attard G., Bidard F.C., Curigliano G., De Mattos-Arruda L., Diehn M., Italiano A., Lindberg J., Merker J.D., Montagut C. (2022). ESMO recommendations on the use of circulating tumour DNA assays for patients with cancer: A report from the ESMO Precision Medicine Working Group. Ann. Oncol..

[239] Lynch F.A., Rodin G., Jefford M., Duffy M., Lai-Kwon J., Heynemann S., Mileshkin L., Briggs L., Burke J., Leigh L. (2023). Evaluation of Managing Cancer and Living Meaningfully (CALM) in people with advanced non-small cell lung cancer treated with immunotherapies or targeted therapies: Protocol for a single-arm, mixed-methods pilot study. BMJ Open.

[240] Temel J.S., Greer J.A., Muzikansky A., Gallagher E.R., Admane S., Jackson V.A., Dahlin C.M., Blinderman C.D., Jacobsen J., Pirl W.F. (2010). Early palliative care for patients with metastatic non-small-cell lung cancer. N. Engl. J. Med..

